# Thermal ages of the Huatung Basin determined from seismic waveform modeling: insights into Southeast Asia’s evolution

**DOI:** 10.1038/s41598-023-42454-x

**Published:** 2023-09-14

**Authors:** Justin Yen-Ting Ko, Ban-Yuan Kuo, Shu-Chuan Lin, Yu-Sheng Hung

**Affiliations:** 1 Institute of Oceanography, National Taiwan University, Taipei, Taiwan; 2 Institute of Earth Sciences, Academia Sinica, Taipei, Taiwan

**Keywords:** Seismology, Geophysics

## Abstract

The Huatung Basin (HB), situated on the leading part of the Philippine Sea Plate, is directly involved in oblique subduction and mountain building in the Taiwan region. However, previous studies have reported a wide range of ages for the HB, from 30 to 130 Ma, making it difficult to properly constrain regional tectonics. We analyzed teleseismic waveforms recorded on Taiwan that traveled through the slab associated with the HB. By waveform matching, we have constrained the slab dimensions to approximately 400 km in length and 150 km in width, accompanied by an enhanced P-wave velocity of 6% within the slab core and an apparent dip angle of 55°. We used age-dependent subduction zone thermal models to estimate the thermal ages or the ages since the last thermal event of the HB. The best-fit thermal model indicates thermal ages ranging from 20 to 50 Ma, which is consistent with a suite of geophysical observations and the age inferred from geomagnetic anomaly data. However, our results differ considerably from the ages obtained through radiometric dating of rocks dredged from the seafloor. The discrepancy in age may be attributed to either thermal rejuvenation of the plate or dating of allochthonous samples dredged from the border of the basin.

## Introduction

**Figure 1:**
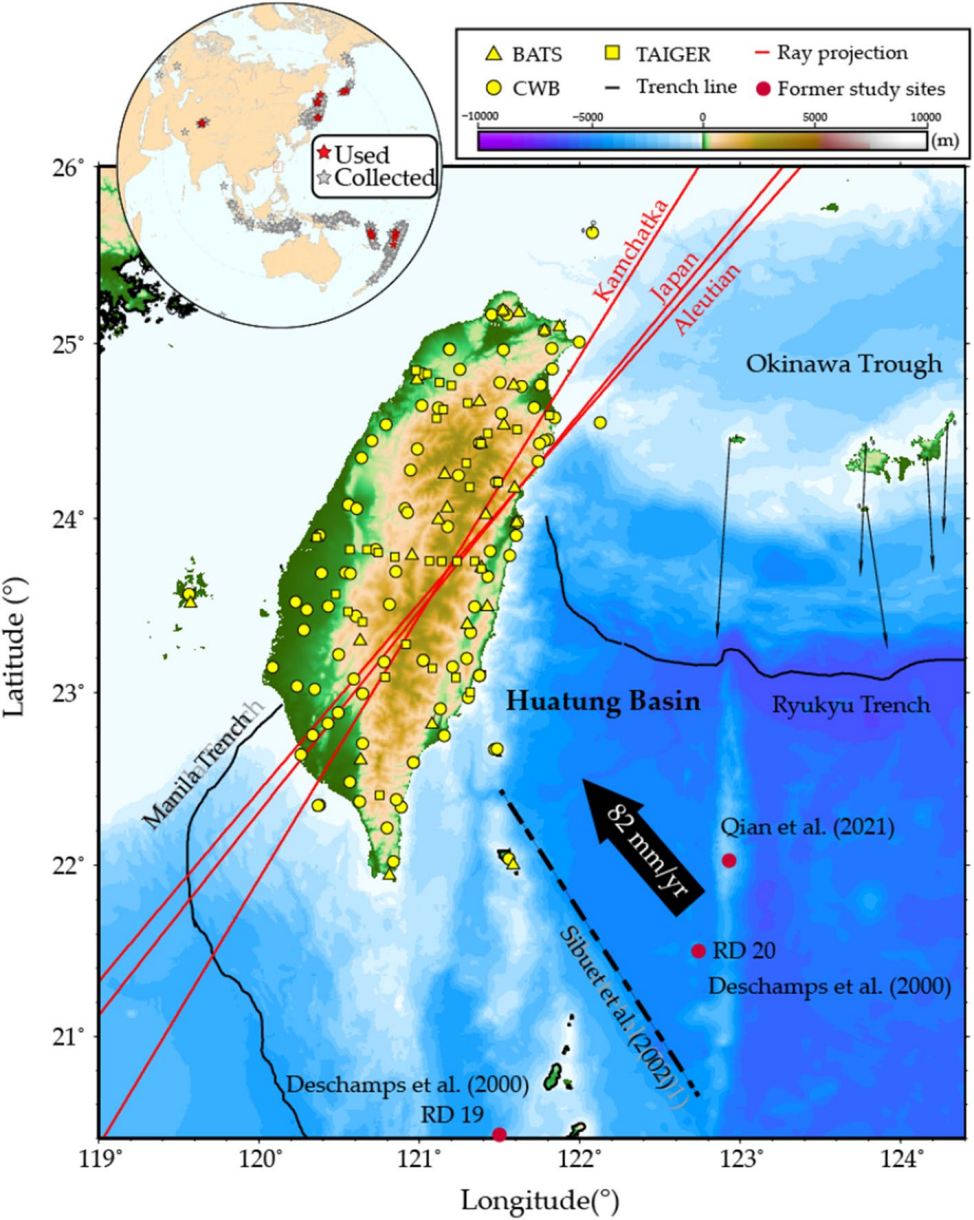
Huatung Basin in the Taiwan collision-subduction zone. The black lines show the locations of the Ryukyu Trench and the Manila Trench. The black lines with arrows indicate the GPS horizontal velocity field with respect to a stable South China^9^. The yellow symbols represent seismic stations. Red dots denote the locations of rock samples dredged from the seafloor for radiometric dating^10,11^. The dashed line constitutes a plate boundary within the HB based on the magnetic anomalies^7^. The inset on the upper left presents the seismic events for which data collection (gray) and processing were conducted (red).

The convergence between the Philippine Sea Plate (PSP) and the Eurasian Plate (EP) resulted in the subduction of the EP beneath the PSP along the Manila Trench, the collision of the Luzon Volcanic Arc with the Eurasian margin, and the subduction of the PSP beneath the Ryukyu Trench (Fig. 1). The part of the PSP intimately involved in all three of these processes is located in the region of the Huatung Basin (HB), the properties of which are therefore critical to the mode of arc-continent collision in Taiwan^1,2^ and to the tectonic evolution of the western PSP^3,4^. The HB has a distinct tectonic identity from the West Philippine Basin (WPB), which is located to the east of the HB on the east side of the Gagua Ridge (Fig. 1). Across the Gagua Ridge, the seafloor of the HB is 600 m shallower than that of the neighboring WPB. According to mantle cooling and seafloor subsidence models^5^, the shallower depth of the HB implies a younger age for the HB than for the WPB, which has an age of approximately 35–56 Ma. Paleomagnetic analyses have indicated a mid- to late-Eocene age for the HB^6,7^. A recent study identified magnetic anomaly chrons 15–19, corresponding to an age range of 35–41 Ma, increasing southward from the Ryukyu Trench to the southern corner of the HB^8^. The age contrast between the HB and the WPB is compatible with the age prediction based on seafloor subsidence^5^.

Even though paleomagnetic modeling of the HB with a limited number of magnetic peaks is subject to uncertainty, a young HB relative to the WPB is favored by wide-angle seismic imaging across the Gagua Ridge^12^ and by thermal models of subduction zones constrained by seismicity^13^. Conversely, radioisotope geochronology has yielded different results. An ^40^Ar/^39^Ar dating of gabbro samples dredged from the HB seafloor indicated an early Cretaceous age of approximately 110–130 Ma^10^. A more recent radiometric dating of igneous rocks dredged from the western flank of the Gagua Ridge provided the same age estimate^11^. The old HB was further suggested by the detailed geochemical analysis of a new set of volcanic rock samples from the peak of the Gagua Ridge. Zhang et al.^14^ revealed that these rocks, also dated to 110–130 Ma, had isotopic signatures linking to typical island arcs in the subduction zone. They hypothesized that the Gagua Ridge was the island arc relic from the oceanic plate subducted under the HB underlain by an early Cretaceous mantle. The consistency in the dating result bolsters the hypothesis of an early-Cretaceous formation of the HB.

Seismology can also provide insights into oceanic lithosphere evolution on the basis that cooling causes an increase in the seismic velocity, a phenomenon well documented across oceanic basins^15,16^. Kuo et al.^17^ conducted a study and discovered that the phase velocities across the HB are similar to those of the Parece Vela Basin^18^, which has a well-defined oldest age of approximately 30 Ma^19^ whereas the two basins coincide in average seafloor depths at ~4800 m (sediment correct would increase the depth of HB relative to that of the Parece Vela Basin by <300 m). These observations led Kuo et al. to the hypothesis that the HB has an age of 20–40 Ma. Another age-varying seismological parameter is the gap between the double seismic layers within a slab, which increases with subduction zone age^20^. Chou et al.^21^ determined a 15–20-km-wide gap across the HB slab, contrasting with the 30–40-km-wide gap in the subducted Pacific Plate beneath northeast Japan^22^, where the plate has an age of 130 Ma. According to the global double-seismic zone model^20^, the HB would correspond to a slab with an age of approximately 50 Ma.

The age estimates derived from geophysical data are likely indicative of tectono-thermal ages, reflecting the time elapsed since the last significant tectonic or thermal events that reset the physical state of the lithosphere^23^. This interpretation contrasts with radiometric dating results from dredged seafloor samples proposing an early Cretaceous age for the HB. This age dichotomy holds considerable significance. In the dynamic model of collision and subduction in the Taiwan region^1^, inclusion of an Eocene-aged HB leads to pronounced rollback of the subducted slab accompanied by shear stress that favors the development of anisotropy compatible with observed SKS splitting patterns^2^, while a 120 Ma old slab would not produce the same effect. In the broad sense, the systematic difference between geophysical and geochemical observations signals a fundamental problem from either or both, and reconciling them may shed new light on the mantle dynamics and tectonic evolution of southeast Asia. However, before a true reconciliation can be realized, such as dating of core samples, both approaches should be continually corroborated or challenged with new data and techniques.

In this study, we investigated the amplitude and waveform anomalies from teleseismic waveform data recorded by seismic stations in Taiwan^24–26^ (Fig. 1) to constrain the slab morphology; we then compared the waveform modeling results with the thermal modeling results, the latter being sensitive to the age of the oceanic basins. This analysis is focused on the slab and could bolster the observations on the basin immediately seaward of the trench^17^. The seismic constraints presented in this study are independent from previous geophysical observations.

## Results

### Amplitude anomalies and multipath effects

**Figure 2:**
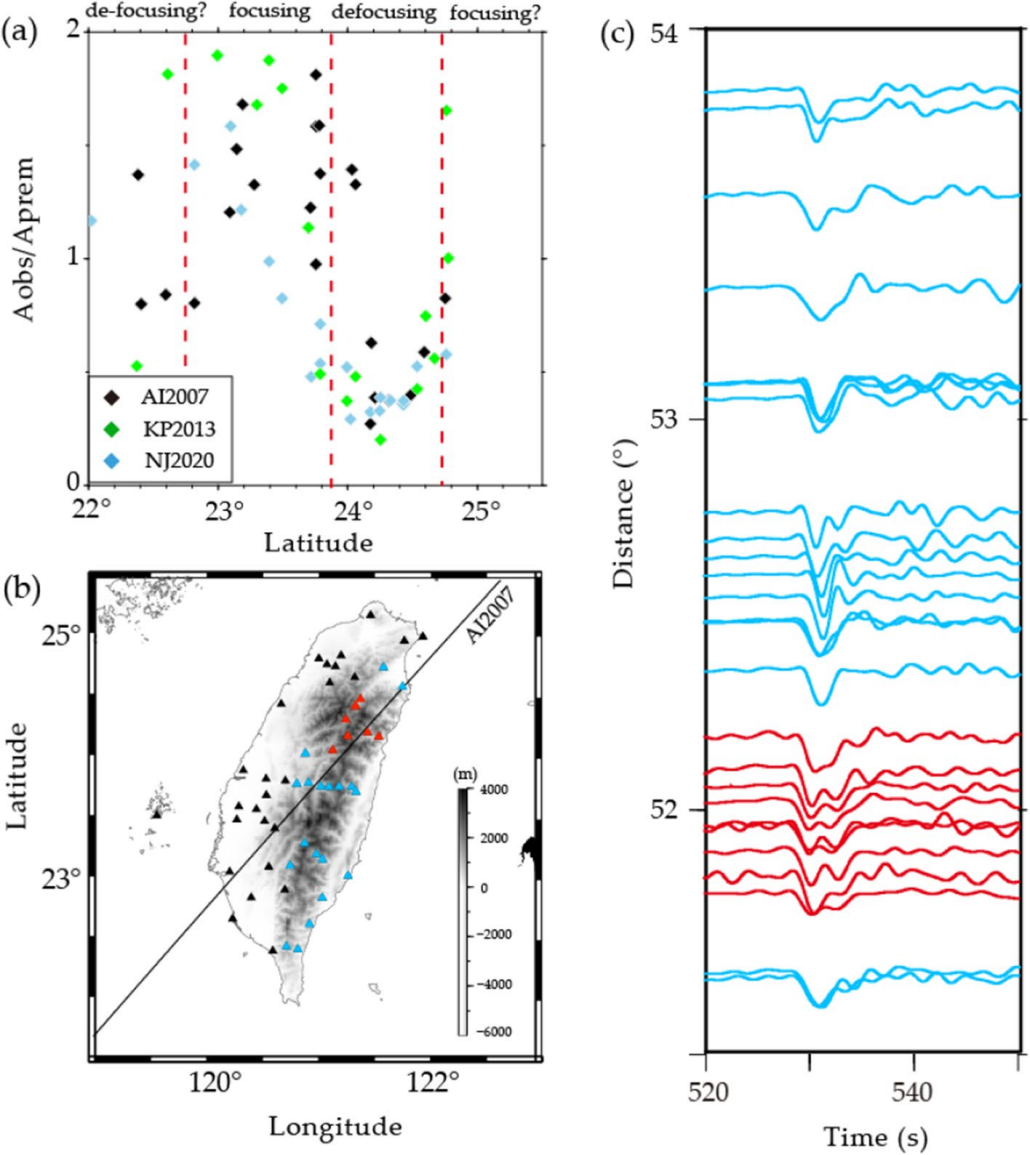
Amplitude measurements, a map view of station distributions, and observed waveform data. (**a**) Combination of the amplitude ratios observed in three events (AI2007, KP2013, NJ2020). Focusing–defocusing effects are observable in certain latitudes (within the red dashed lines), especially for AI2007. (**b**) Spatial distribution of the seismic stations (triangles) for AI2007. The red triangles represent the stations whose data display clear double arrivals. The blue triangles represent the stations plotted in (**c**) without apparent multipath appearances, and the black triangles represent the stations whose data were not used. (**c**) The displacement waveform section of AI2007 shows clear multipath arrivals of P waves near 52° (red traces). The waveforms were subjected to band-pass filtering with cutoff frequencies of 0.03 to 0.5 Hz.

The analysis of P wave amplitude anomalies necessitates a nuanced approach to focusing-defocusing effects. When encountering high-velocity anomalies, wavefront behavior can lead to either amplitude enhancement through ray focusing or attenuation through ray defocusing, contingent upon structural geometry and seismic ray trajectories. To measure relative amplitudes, we cross-correlated the P wave between observed and synthetic waveforms computed using the finite difference (FD) method^27^. Our analysis revealed that the amplitude anomalies of the P wave recorded from different events were largely consistent (Fig. 2). Taking the event from Aleutian (AI2007) for example, a distinctive U-shaped distribution of amplitude measurements becomes evident in the latitude range of 23° to 25° (Fig. 2a). This striking pattern in amplitude values suggests the potential influence of high-attenuation structures or significant wavefield distortions, which are often associated with multipathing effects (Figs. 2b and c). However, it is essential to note that the presence of strong attenuation structures becomes less likely, given the concurrent observation of low amplitudes in conjunction with fast travel-time patterns (Extended Data Fig. 2). Previous waveform modeling studies^28,29^ suggested that observed waveform complexities may imply the wavefiled passing through the sharp velocity structures which cannot be explain by tomographic model. An investigation was conducted here to assess the ability of tomographic model of the TX2019slab^30^ to explain the observed anomalies. Notably, the TX2019slab has already incorporated a priori 3-D subducting slabs in their starting model. It was found that while the TX2019slab partially accounted for the travel-time variations, they failed to predict the anomalies observed in amplitude and waveform characteristics (Extended Data Fig. 1). Even when considering stronger heterogeneities within the tomographic model, it still could not adequately capture the distinctive features exhibited by the observed data (Extended Data Fig. 1). Therefore, our findings highlight the significance of incorporating sharp velocity variations within the model to accurately account for the observed seismic observations.

### Determination of the optimal slab model

**Figure 3:**
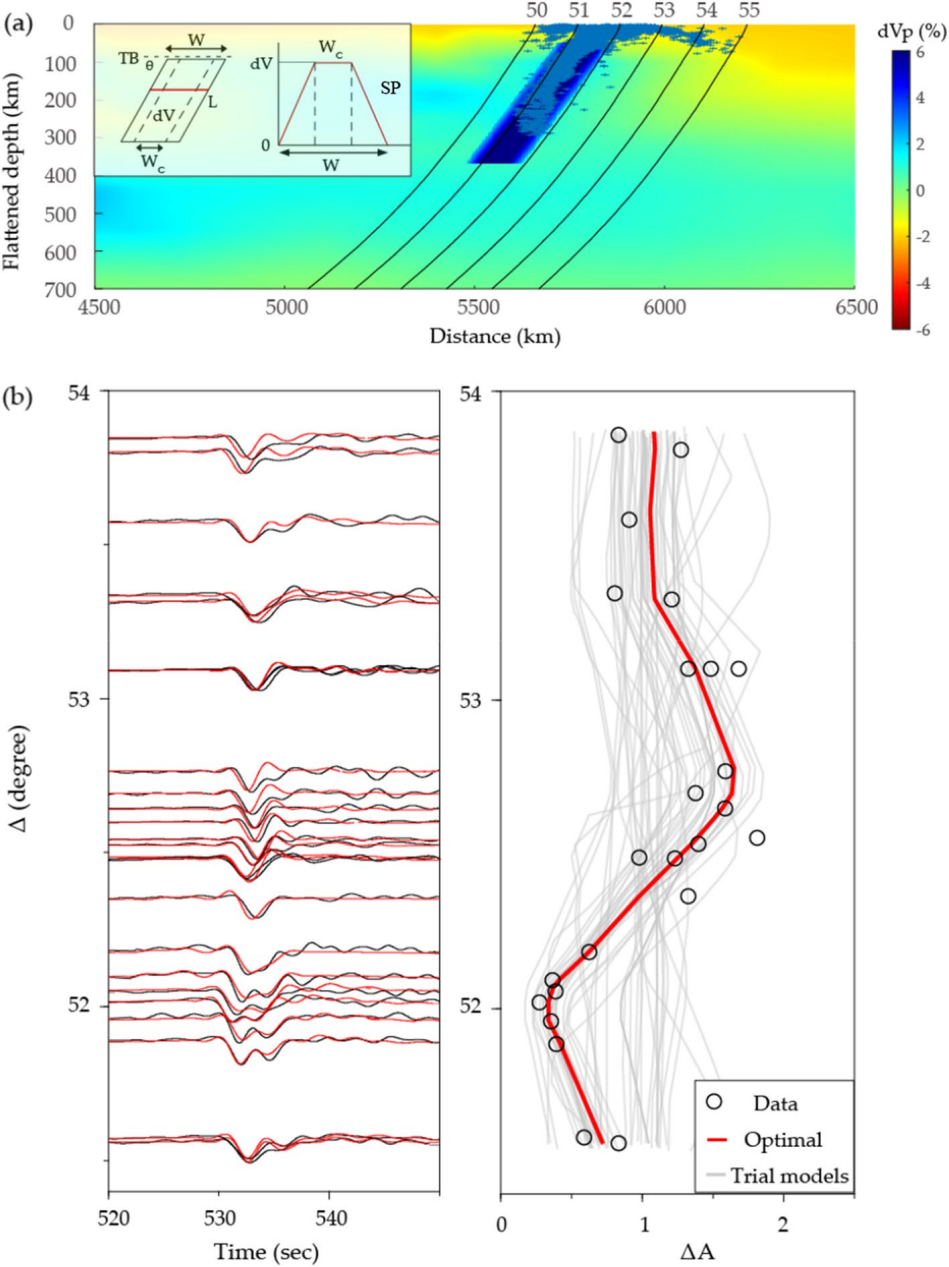
Amplitude ratio and waveform fitting of the optimal slab parameter for AI2007. (**a**) Optimal slab model embedded into TX2019 slab, with ray paths representing different epicentral distances. Blue dots represent the background seismicity. The inset on the left presents the model parameters used to characterize the idealized slab, including top boundary (TB), slab dip ($\uptheta$), slab width (W), width of the slab core (W_C_), and velocity perturbation within the slab core ($\delta \mathrm{V}$). The inset on the right shows the velocity structures within the idealized slab model. (**b**) Fitting results for waveforms (left) and amplitudes (right). Red indicates predictions made by the optimal model. Black represents the data. The gray lines in the right panel indicate the randomly selected trial models.

To model the observed amplitude anomalies and waveform shapes for AI2007, we developed a two-dimensional slab model that was embedded into the TX2019 slab background model^30^. This slab model was assumed parallelogram in shape and characterized by six parameters, including top boundary (TB), length (L), width (W), dip angle ($\uptheta$) of the slab, velocity perturbation ($\delta \mathrm{V}$) relative to PREM model within the slab core, and slab sharpness (SP) which represents the absolute value of velocity decay rate from the boundary of the slab core to the slab edge (Fig. 3a). We acknowledge that the assumption of identical sharpness for both slab edges depart from what thermal models predict. However, we opted for a simplified model given that data constraints on the top boundary do not merit a separate parameter (Fig. 3a). The parallelogram block is inserted into the TX2019slab model, and the velocity fluctuations are replaced with our idealized velocity structures inside the block. By incorporating these parameters into the model, a more comprehensive understanding of the slab morphology was achieved. Using a GPU-based FD method^27^, we generated a library of approximately 6000 Green’s functions for numerous slab models. To constrain the horizontal location of the slab, we utilized the relocated background seismicity^31^. By performing a grid search for model parameters, we minimized the differences in amplitudes and waveform shapes between real and synthetic data^29,32^. Our modeling results indicate that the slab has a length of approximately 400 km (L), a width of 150 km (W), an enhanced P wave velocity of 6% within the slab core ($\delta \mathrm{V}$), and an apparent dip angle of 55° (θ) (Extended Data Fig. 8). A core with a width of 60 km (W_C_), flanked by edges that taper on either side and are 45 km thick, provides the optimal fit for the waveform data, accounting for both amplitude anomalies and double arrivals observed (Fig. 3b). The best-fit SP value is 0.133 1/km. This model not only fits the measurements for the studied event, but also explains the data from two other events, KP2013 and NJ2020 (Extended Data Fig. 10).

### Uncertainties of model parameters

The suite of sensitivity tests conducted as outlined in the Method section and depicted in Extended Data Figs. 3 to 8 underscored the convergence of discrete parameters in influencing both amplitudes and waveform responses. The inherent trade-offs that emerge between model parameters were found to be intrinsic within the ambit of exploring the intricate five-parameter model space. Our subsequent inversion findings assert that larger velocity perturbations necessitate the incorporation of smaller slab lengths to attain a harmonious data fit, a point exemplified in Extended Data Fig. 9a. Through meticulous analysis, while factoring in a range of uncertainties of around 5%, it becomes evident that velocity perturbations gravitate towards a spectrum of 5% to 7%, concurrently accompanied by slab lengths spanning 450 km to 260 km. With an integrated consideration of both slab dip and length variances, the range of uncertainty regarding slab length is effectively constrained within 330 km to 420 km (Extended Data Fig. 9b). In a relative comparison, it becomes discernible that in relation to velocity perturbation and slab dip, which manifest a relatively substantial range of variations, the determined parameters of slab width and sharpness exhibit enhanced precision (Extended Data Fig. 9d). While the model with a width of 100 km and a sharper gradient could also provide a good fit to the data (Extended Data Fig. 9d), the seismicity distribution depicted in Fig. 3a supports the notion that a slab width of 150 km is more reasonable. Our inversion outcomes compellingly delineate that the slab width remains confined to a range of 147 km to 160 km, while the slab sharpness is constrained within the bounds of 0.11 to 0.15 1/km. Given the notably robust constraint achieved concerning slab width and sharpness, we have subsequently channeled the refined estimates of these two parameters in the subsequent age estimation of the respective slab ages.

### Inferring the thermal age of the HB slab

As emphasized above, we determine the “tectono-thermal ages”^23^ which may or may not represent the age of the formation of the basin^33^. To simplify the terminology, we will refer to these ages as "thermal ages" because the thermal effect is critical to our interpretations (as explained below), or simply as "ages" when the context permits. To determine the thermal age of the PSP slab, we compared our optimal seismic slab model with subduction zone thermal models calculated using well-established techniques^9,34–36^. The detailed descriptions of these thermal models are provided in the Method section.

**Figure 4:**
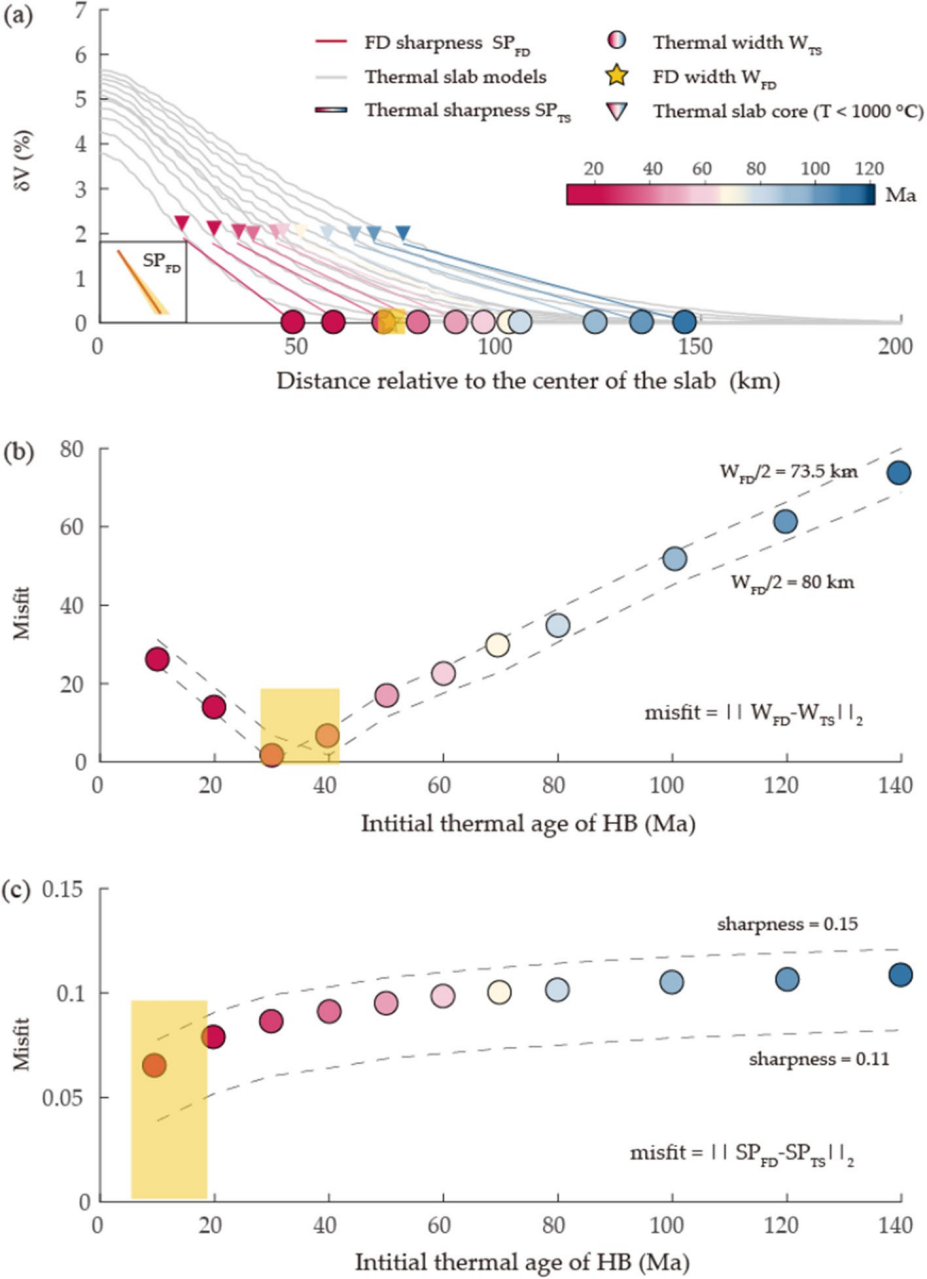
Age determination. (**a**) The thermal slab models yielded $\delta \mathrm{V}$ values, with the gray lines indicating the predictions obtained from age-dependent models. The left-to-right sequence of initial thermal ages for HB are 10, 20, 30, 40, 50, 60, 70, 80, 100, 120, and 140 Ma, respectively. The color-coded lines indicate the linear regression results of $\delta \mathrm{V}$ values ranging from the edge of the thermal slab core (color-coded triangles) to 0.1. The yellow star and color-coded circles indicate the estimated width of the slab from seismic and thermal slab models, respectively. The inset represents the slab sharpness (red line) obtained by seismic slab model. The shaded yellow zones depict the uncertainties of the slab thickness and sharpness, within which the selected parameters varied less than 5% from their optimal values. (**b**) Misfit of slab width between the seismic and thermal slab models at different ages (color coded). (**c**) Misfit of sharpness between the seismic and thermal slab models at different ages (color coded).

In the thermal modeling, we fixed the dip and length of the slab at the optimal values given by the waveform modeling, i.e., 55° and approximately 400 km (see below), respectively. The subduction rate was fixed at 55 mm/year, as projected from the plate convergence rate of 82 mm/year^4^. We generated a series of thermal models with initial ages of 10–140 Ma for the subducting plate and fixed ages of 50 Ma and 80 Ma for the overriding plate. In this series of models, the subduction duration is fixed at 7.3 My which yielded a slab subducted with a down-dip length from the surface approximately 400 km. Subsequently, we employed a temperature derivative of − 0.5%/100K^37,38^ to convert the temperatures into corresponding velocity perturbations. The estimation of the thermal slab’s width, denoted as W_TS_, and its sharpness, designated as SP_TS_, was conducted as follows. In a cross-sectional plane perpendicular to the slab and positioned at a depth of 150 km, the temperature-derived velocity exhibits its peak value at the center of the thermal slab, marked by the coordinate x=0 (Fig. 4a). It is noteworthy that variations in the temperature derivative used primarily influence the strength and sharpness of the slab (Extended Data Fig. 11), while inducing negligible modifications to the overall width of the slab and ages determination, as evidenced in Extended Data Fig. 12. To align our thermal slab model with the FD model, we defined the slab core boundary at 1000 °C. Within this core, we assumed a constant dV_TS_ (Extended Data Fig. 13e). We determined the half width of the thermal slab by fitting a linear slope to the velocity decrease from the boundary of the thermal slab core to 0.1 and extrapolating it to zero on the x-axis. The thermal slab sharpness, SP_TS,_ was calculated as the ratio of $\delta \mathrm{V}$_TS_ to the distance spanned from $\delta \mathrm{V}$_TS_ to zero. Accounting for the inherent uncertainties associated with the width of seismic slab (W_FD_), the observed variation in width between the seismic slab model and the thermal slab model (W_TS_), within the context of age, highlights a noteworthy nadir at initial model ages of approximately 30–40 Ma (as depicted in Fig. 4b). Intriguingly, this age range aligns with true ages of approximately 40–50 Ma for the subduction process of HB spanning 7.3 Ma. When incorporating the intrinsic uncertainties affiliated with the sharpness of seismic slab (SP_FD_), the misfit in sharpness unveils a tantalizing possibility: the inception ages of the PSP slab could plausibly range from as young as 10 Ma, or approximately 20 Ma post-subduction (Fig. 4c). The adoption of a lower core temperature, specifically 650 °C, yields the optimal fitting initial ages at 40 Ma for W_TS_ and 10 Ma for SP_TS_. These estimates correspond to true ages of approximately 50 and 20 Ma, respectively. Notably, the ascertained late Eocene age is in concurrence with earlier assessments attained through diverse methodologies such as shear velocities^17^, the double seismic zone^21^, thermal models^13^, and magnetic anomalies^6–8^. Conversely, attributing an early Cretaceous age^10^ to the slab would engender an excessively thick slab configuration, failing to align with the discerned sharp velocity gradient and thickness of the slab deduced from waveform modeling. Furthermore, our analysis gains additional support from synthetic assessments directly involving thermal models (Extended Data Fig. 14). Although these models may not precisely replicate the data alignments achieved by our FD model, they provide informative results. We assessed the thermal slab’s thickness based on its velocity perturbations, measuring the distance between the top and bottom boundaries where δV=0 (Extended Data Fig. 14a). While a 150 km thickness may appear too thick for a 40 My old slab, it corresponds approximately to a 100 km thick slab if we define the slab boundary by a temperature of 1200 °C. These findings reinforce the notion that assigning an early Cretaceous age to the slab would result in an excessively thick slab configuration, which fails to adequately match the amplitude measurements (Extended Data Fig. 14b).

A related source of uncertainty emanates from the assumptions inherent in our thermal modeling strategy. Our approach entails deriving the initial temperature from a half-space cooling model (HS) at a designated age. However, we recognize that this method could potentially introduce a bias towards cooler mantle conditions when compared to predictions based on seafloor depth flattening and heat flow at old ages^39^. To address this potential bias on our conclusions, we constructed alternative subduction thermal models. These models adopt initial temperatures from the plate model (PM), which predicts the subsidence and heat flow data more reasonably than the HS at old seafloor^39^ (Extended Data Fig. 15). In a study by Richards et al.^40^, a conventional PM using a constant thermal conductivity yielded satisfactory data fitting with a plate thickness of 105 km. The optimal agreement, however, emerges by integrating temperature-dependent conductivity and a 135 km thick plate. To account for the range of uncertainty in plate thickness, we considered these two values in our analysis. As shown in Extended Data Fig. 12, the ages best fitting the 105 km- and 135 km-PM models consistently fall within the 30–50 Ma range, aligning with our original findings. This agreement arises naturally for the 135 km-PM, while it remains unsurprising for a 105 km plate because the thermal structures between the two types of models deviate substantially only after 80 Ma^40^ , which is well beyond the range of the minimum misfit observed at 30–50 Ma.

## Discussion

Mounting geophysical evidence for a young, Eocene HB prompts speculation concerning what factors may be contributing to the contradictory age estimations, whether they are the formation ages or thermal ages. One possible scenario is that rocks dredged from the seafloor without drilling into the basement may be allochthonous and nonrepresentative of the in-situ properties of the HB. Note that all the dated samples were collected along the border of the HB. In this scenario, crustal fragments of an old plate need to be within reasonable reach to be dredged. Sibuet et al.^7^ argued that the samples dated to the early Cretaceous^10^ were dredged from the southernmost HB, where relics of old, different basins may remain on the seafloor. Conversely, the younger geophysical ages were inferred from observations made in the middle or northern part of the HB^17^ or within the subducted slab^13,21^. One candidate for the unknown older plate in the region could be the proto-South China Sea Plate, which is a hypothetical basin that was entirely consumed by early to mid-Cenozoic subductions^41–43^. This hypothetical plate could be Mesozoic in age^42^, although it is essentially unconstrained.

In contrast to the allochthon scenario, the autochthon scenario proposes that the dredged samples^10^ represent in situ basement rocks of the HB, leading to the hypothesis that the lithosphere of the HB has undergone thermal rejuvenation, making it appear younger than its actual age. Kuo et al.^17^ argued that a Hawaiian-like plume would be necessary to lift an early Cretaceous basin to the level of the present-day HB. Note that the plume-driven uplift could be reduced from 1.3^17^ to 1 km if the seafloor stopped subsiding at 70–80 Ma^44^. However, the 1-km elevation still falls within the range of the elevations of the Hawaiian swell relative to the surroundings. It appears that a strong plume is still required to compensate the age-dependent subsidence of an early Cretaceous HB.

Tectono-thermal events have been documented to alter the lithospheric properties to the extent that they mask the true ages of the lithosphere. For example, some Archean cratons that have been reworked by Phanerozoic tectono-magmatic events demonstrate thermal thicknesses equivalent to the lithosphere of ~1 Ga^33^. The elastic thickness of the continental plate often shows a positive correlation with the thermal ages of different geological provinces^45,46^. In the Hawaiian hotspot swell region, the Pacific plate is elevated to depths corresponding to the seafloor of 25 Ma^47^. Seismological imaging suggests that the Hawaiian plume may have thinned the Pacific plate by thermally altering the lower half of the plate^48,49^. However, a large-scale thermal event beneath the present-day HB has yet to be identified. Localized events, such as a drastic thermal overturn stirred by possible slab detachment beneath central Taiwan^1^, are considered less capable of rejuvenating the HB. Further investigation is needed to explore the hypothesis of thermal rejuvenation of the HB lithosphere in the autochthon scenario.

Here we examine possible thermal sources in the broad western Pacific. Based on the geochemistry of an IODP basement core, Hickey-Vargas et al.^50^ proposed that upwelling of the mantle may have existed beneath the PSP predating the magmatism that created the Izu–Bonin–Mariana arc. However, the drilling site was located north of the Daito Ridge, making it difficult to link to the HB. The model of Gagua Ridge as a relict volcanic arc^14^ mentioned above suggests that the HB once overrode a mantle wedge and therefore was susceptible to extra heating. Whether these asthenospheric upwelling^50^ or the mantle wedge^14^ processes were vigorous enough to rejuvenate an early Cretaceous basin to a thermal age of Eocene remains to be explored. One hypothesis is that, along its evolutionary path, the HB lithosphere was heated and thinned by a large-scale mantle plume and thermally reset before it arrived at its present location, where geophysical investigations were conducted on the rejuvenated version of the old HB. We suggest searching for possible thermal events along the long trajectory of the HB from near the equator to Taiwan.

## Conclusions

We present a novel method of using amplitude ratio and P-wave waveform distortions to constrain the morphology of the PSP slab subducted from the HB. Our inversion of these data produced an optimal slab model, featuring a 400-km-long, 150-km-wide parallelogram with a 55° apparent dip. The high-velocity core exhibited a 6% velocity perturbation increase relative to the PREM model, with a sharp velocity gradient towards both slab edges. A comparison with subduction zone thermal models suggested that the age of the slab, or the age since the last major thermal event is less than 50 Ma. This thermal age estimate adds to the geophysical evidence supporting an apparently young HB in contrast to the postulation of an early Cretaceous HB obtained from the radiometric dating of rocks dredged from the ocean floor. The different age estimates may be reconciled by one of two scenarios: either the seafloor samples dated to the early Cretaceous are allochthonous or the lithosphere of the HB was thermally rejuvenated at some point during its evolution.

## Method

### Data process and measurements

Data from a seismic network consisting of 136 stations between January 2005 and December 2020 were utilized in this study. Earthquake events were selected based on their focal depth being greater than 100 km, their magnitude ranging from 6 to 7, and their epicentral distances between 25° to 90°. Only high-quality displacement waveforms meeting a signal-to-noise ratio threshold of >7 were used, which was defined as the maximum amplitude of the P wave divided by the root mean square of background noise within a 30-second window prior to the P wave arrival. The waveforms were deconvolved to remove the instrument response and subjected to band-pass filtering (Butterworth) with cutoff frequencies of 0.03 to 0.5 Hz. A total of 18 events were included in the final dataset, consisting of three high-quality events from the north-northeast of Taiwan (namely, the Aleutian Islands event in 2007, the Kamchatka Peninsula event in 2013, and the Northeastern Japan event in 2020), as well as 15 events from other azimuthal directions (Extended Data Table 1 and Extended Data Figs. 18–21).

### Waveform modeling and sensitivity

We conducted sensitivity tests on six parameters to assess their effects on the amplitude and waveform characteristics of idealized slab models. The tests involved sequentially altering a single parameter while holding the remaining parameters constant. Our reference slab model was embedded into PREM, and we assumed a width of 150 km, a length of 400 km, a dip angle of 55°, and a slab edge sharpness of 30%, with a 6% increase in P wave velocity. Extended Data Figs. 3–7 depict the results of the sensitivity tests for the AI2007 event, comparing the amplitude and waveforms of P waves among the models. Our findings indicate that increasing the $\delta \mathrm{V}$ of the slab significantly magnified the focusing-defocusing effects on amplitude measurements and led to substantial wavefield distortion, resulting in multiple arrivals. Altering the top-side position, dip angle, and slab width caused horizontal shifts in amplitude anomalies, with changes in the dip angle exerting strong effects on waveform shapes. Additionally, an increase in slab length enhanced focusing-defocusing effects and generated more pronounced multiple arrivals on waveforms. Another crucial parameter was the sharpness of the slab edge, expressed as the rate of the decline in velocity from the boundary of the slab core to the slab edge. To explore the effects on waveforms and amplitudes of changing the sharpness parameter, we extended the slab width to 300 km. As the sharpness increased, the velocity gradient near the slab edge increased, resulting in more notable effects on waveforms (Extended Data Fig. 8).

### Model parameters and grid search

We found that the observed amplitude distribution was accurately mirrored only by incorporating slab models with a minimum depth of 50 km into the reference model, according to sensitivity testing (Extended Data Fig. 3). To further refine our analysis, we conducted a grid search for the remaining five parameters, namely the width (w), length (l), dip angle (θ), velocity perturbation ($\delta \mathrm{V}$), and slab edge sharpness (sp), by minimizing differences in amplitudes and waveform shapes between the actual and synthetic data^26,28^

1 $${\varepsilon }_{t}=\sum_{i=1}^{n}\Vert \frac{\Delta {A}_{i}}{{\sigma }_{\Delta A}}\Vert +\sum_{i=1}^{n}\Vert \frac{1-corr(data,syn)}{{\sigma }_{\mathrm{DC}}}\Vert$$

where *i* and *n* are the index number and total number of data traces, respectively. The first term in the cost function represents the misfit in amplitude measurements. The second term accounts for the waveform residual determined by the waveform dissimilarity between the observed and synthetic wavelets. Each numerator in the cost function is dimensionless following normalization with their standard deviations, which are σ_ΔA_ and σ_DC_, respectively. Taking AI2007 as an example, we present the optimal slab model embedded into the TX2019 slab (Fig. 3a) and its background seismicity. Combining amplitude and waveform decorrelation in the cost function indicated that our fitting strategy can reasonably and effectively reduce the trade-offs between the model variables (Extended Data Fig. 9). Inversion modeling reveals that embedding a thin slab with sharp edges fit the amplitude anomalies well and results in a favorable fit on waveforms (Fig. 3b).

Although our optimized solution appears to be satisfactory in fitting the observed seismic data, it is important to note that the simplifying assumptions made during the modeling process may introduce some level of bias. To better capture the intricate structure of the slab, it is necessary to perform fully three-dimensional simulations. However, due to the significant computational costs associated with searching the multidimensional parameter space, the use of idealized slab models with a single dip angle is justified. Incorporating bending slab models^19^ would substantially increase simulation time, hence justifying our approach. Additionally, the effects of site-specific amplification cannot be ignored as they may contribute to the amplitude anomalies observed. Nonetheless, through comparisons with other events of varying azimuths, it is evident that local structures have minimal influence on the focusing-defocusing patterns observed. For detailed information, refer to the Supplementary Information document.

### Thermal models

We utilized a well-established methodology for modeling the thermal structures of subduction zones, which has been successful in explaining observations and offering insights into the evolution of subduction zones^36,51^. Our model setup involves a steady plate situated above a dynamic mantle wedge, with the wedge flow being driven by subduction of a kinematic slab that has prescribed geometry and velocity^9,36,51^. We employed a finite-volume code^52^ that has contributed to the community benchmark for subduction zone modeling^9^ and has undergone improvements for calculations of non-Newtonian flow after the benchmark work^1,34^.

The model dimensions used were 660 km width and 600 km depth, with a reference mantle temperature of 1350 °C. The horizontal grid spacing (dx) was set at a constant value of 0.9375 km, while the vertical grid spacing (dz) ranged from ~0.25 km at shallow depths to ~2.58 km near the bottom boundary, using dz = dx*atan(θ) that depends on the local angle of the slab (θ). We adopted a fixed slab dip of 55° as constrained by the seismic slab model, a subduction rate of 55 mm/yr, and a subduction duration of 7.3 My, which allowed the slab to attain a length of 400 km, as required by the best-fit seismic slab model. The prescribed thickness of the rigid fore-arc lithosphere was set at 70 km^34^. The initial temperature of the subducting plate was determined by its age, while that of the overriding plate was fixed at 50 and 80 Ma. No transition in temperature was imposed across the subduction interface, and the temperature was allowed to evolve from its initial condition. Regarding the boundary conditions, the temperature was fixed only at the top boundary, and a zero temperature gradient was assumed at other boundaries. In practice, the trench was located at the vertical boundary of the model. For a full scale model, the overriding-plate side of the model was extrapolated from the temperatures at this vertical boundary. Details regarding the heat transfer, mass conservation, and momentum conservation equations for an incompressible Boussinesq fluid, the non-Newtonian rheology, the numerical techniques, physical parameters, and treatments of boundary conditions, were described in Lin et al.^34^.

### Supplementary Information

Supplementary Information.

## Data Availability

All waveform data listed in Extended Data Table 1 are archived and openly available at the TEC Data Center (https://tecdc.earth.sinica.edu.tw/tecdc/). Researchers can register for an account to apply for the data. The figures presented in this work are made by the generic mapping tools (GMT)^53^.

## References

[1] Lin SC, Kuo BY (2016). Dynamics of the opposite-verging subduction zones in the Taiwan region: Insights from numerical models. J. Geophys. Res. Solid Earth.

[2] Kuo BY, Lin SC, Lin YW (2018). SKS splitting and the scale of vertical coherence in the Taiwan mountain belt. J. Geophys. Res. Solid Earth..

[3] Wu J, Suppe J, Lu R, Kanda R (2016). Philippine Sea and East Asian plate tectonics since 52 Ma constrained by new subducted slab reconstruction methods. J. Geophys. Res. Solid Earth.

[4] Sibuet J-C, Zhao M, Wu J, Lee C-S (2021). Geodynamic and plate kinematic context of South China Sea subduction during Okinawa trough opening and Taiwan orogeny. Tectonophysics.

[5] Parsons B, Sclater JG (1977). An analysis of the variation of ocean floor bathymetry and heat flow with age. J. Geophys. Res..

[6] Hilde TWC, Lee C-S (1984). Origin and evolution of the West Philippine Basin: A new interpretation. Tectonophysics.

[7] Sibuet J-C, Hsu S-K, Le Pichon X, Le Formal J-P, Reed D, Moore G, Liu C-S (2002). East Asia plate tectonics since 15 Ma: Constraints from the Taiwan region. Tectonophysics.

[8] Doo WB, Hsu SK, Yeh YC, Tsai CH, Chang CM (2015). Age and tectonic evolution of the northwest corner of the West Philippine Basin. Mar. Geophys. Res..

[9] van Keken PE, Wada I, Abers GA, Hacker BR, Wang K (2018). Mafic high-pressure rocks are preferentially exhumed from warm subduction settings. Geochem. Geophys. Geosyst..

[10] Deschamps A, Monié P, Lallemand S, Hsu S-K, Yeh KY (2000). Evidence for early Cretaceous oceanic crust trapped in the Philippine Sea Plate. Earth Planet. Sci. Lett..

[11] Qian S, Zhang X, Wu J, Lallemand S, Nichols ARL, Huang C, Miggins DP, Zhou H (2021). First identification of a Cathaysian continental fragment beneath the Gagua Ridge, Philippine Sea, and its tectonic implications. Geology.

[12] Eakin DH, McIntosh KD, Van Avendonk HJA, Lavier L (2015). New geophysical constraints on a failed subduction initiation: The structure and potential evolution of the Gagua Ridge and Huatung Basin. Geochem. Geophys. Geosyst..

[13] Gutscher M-A, Klingelhoefer F, Theunissen T, Spakman W, Berthet T, Wang TK, Lee C-S (2016). Thermal modeling of the SW Ryukyu forearc (Taiwan): Implications for the seismogenic zone and the age of the subducting Philippine Sea Plate (Huatung Basin). Tectonophysics.

[14] Zhang G, Zhang J, Dalton H, Phillips D (2022). Geochemical and chronological constraints on the origin and mantle source of Early Cretaceous arc volcanism on the Gagua Ridge in western Pacific. Geochem. Geophys. Geosys..

[15] Nishimura CE, Forsyth DW (1989). The anisotropic structure of the upper mantle in the Pacific. Geophys. J. Int..

[16] Richards FD, Hoggard MJ, White N, Ghelichkhan S (2020). Quantifying the relationship between short-wavelength dynamic topography and thermomechanical structure of the upper mantle using calibrated parameterization of anelasticity. J. Geophys. Res. Solid Earth.

[17] Kuo B-Y, Chi W-C, Lin C-R, Chang ET-Y, Collins J, Liu C-S (2009). Two-station measurement of Rayleigh-wave phase velocities for the Huatung basin, the westernmost Philippine Sea, with OBS: Implications for regional tectonics. Geophys. J. Int..

[18] Isse T, Shiobara H, Fukao Y, Mochizuki K, Kanazawa T, Sulgioka H, Kodaira S, Hino R, Suetsugu D (2004). Rayleigh wave phase velocity measurements across the Philippine sea from a broad-band OBS array. Geophys. J. Int..

[19] Sdrolias M, Roest WR, Muller RD (2004). An expression of Philippine Sea plate rotation: the Parece Vela and Shikoku Basins. Tectonophysics.

[20] Brudzinski MR, Thurber CH, Hacker BR, Engdahl ER (2007). Global prevalence of double Benioff zones. Science.

[21] Chou HC, Kuo BY, Hung SH, Chiao LY, Zhao D, Wu YM (2006). The Taiwan-Ryukyu subduction-collision complex: folding of a viscoelastic slab and the double seismic zone. J. Geophys. Res..

[22] Shiobara H, Sugioka H, Mochizuki K, Oki S, Kanazawa T, Fukao Y, Suyehiro K (2010). Double seismic zone in the North Mariana region revealed by long-term ocean bottom array observation. Geophys. J. Int..

[23] Artemieva IM (2009). The continental lithosphere: Reconciling thermal, seismic, and petrologic data. Lithos.

[24] Institute of Earth Sciences, Academia Sinica, Taiwan Broadband Array in Taiwan for Seismology. Institute of Earth Sciences, Academia Sinica, Taiwan. Other/Seismic Network. 10.7914/SN/TW (1996).

[25] Central Weather Bureau (CWB, Taiwan). Central Weather Bureau Seismographic Network. Int. Fed. Dig. Seismogr. Netw. 10.7914/SN/T5 (2012).

[26] Okaya D, Wu F, Avendonk HV (2006). Taiwan Supplement #2 to TAIGER project. Int. Fed. Dig. Seismogr. Netw..

[27] Li D, Helmberger DV, Clayton R, Sun D (2014). Global synthetic seismograms using a 2D finite-difference method. Geophys. J. Int..

[28] Sun D, Helmberger D (2011). Upper-mantle structures beneath USArray derived from waveform complexity: Upper-mantle structures beneath USArray. Geophys. J. Int..

[29] Ko JY-T, Helmberger DV, Wang H, Zhan Z (2017). Lower mantle substructure embedded in the Farallon plate: The Hess conjugate: Hess conjugate embedded in Farallon slab. Geophys. Res. Lett..

[30] Lu C, Grand SP, Lai H, Garnero EJ (2019). TX2019slab: A new P and S tomography model incorporating subducting slabs. J. Geophys. Res. Solid Earth.

[31] Wu YM, Chang CH, Zhao L, Teng TL, Nakamura MA (2008). Comprehensive Relocation of Earthquakes in Taiwan from 1991 to 2005. Bulletin Seismol. Soc. Am..

[32] Ko JY-T, Hung S-H, Kuo B-Y, Zhao L (2017). Seismic evidence for the depression of the D” discontinuity beneath the Caribbean: Implication for slab heating from the Earth’s core. Earth Planet. Sci. Lett..

[33] Artemieva IM (2006). Global thermal model TC1 for the continental lithosphere: Implications for lithosphere secular evolution. Tectonophysics.

[34] Lin SC, Kuo BY, Chung SL (2010). Thermomechanical models for the dynamics and melting processes in the Mariana subduction system. J. Geophys. Res..

[35] van Keken PE, Currie C, King SD, Behn MD, Cagnioncle A, He J, Katz RF, Lin S-C, Parmentier EM, Spiegelman M, Wang K (2008). A community benchmark for subduction zone modeling. Phys. Earth. Planet. Int..

[36] Hao M, Li Y, Zhuang W (2019). Crustal movement and strain distribution in East Asia revealed by GPS observations. Sci Rep.

[37] Karato SI (1993). Importance of anelasticity in the interpretation of seismic tomography. Geophys. Res. Lett..

[38] Cammarano F, Goes S, Vacher P, Giardini D (2003). Inferring upper-mantle temperatures from seismic velocities. Phys. Earth Planet. Inter..

[39] Turcotte, D.L. & Schubert, G. Geodynamics, Cambridge University Press, Cambridge. 10.1017/CBO9780511807442 (2002).

[40] Richards FD, Hoggard MJ, Cowton LR, White NJ (2018). Reassessing the thermal structure of oceanic lithosphere with revised global inventories of basement depths and heat flow measurements. J. Geophys. Res. Solid Earth.

[41] Cullen AB (2010). Transverse Segmentation of the Baram-Balabac Basin, NW Borneo: Refining the model of Borneo’s tectonic evolution. Petro. Geosci..

[42] Hall R, Breitfeld HT (2017). Nature and demise of the Proto-South China Sea. Geo. Soc. Malaysia Bull..

[43] Wu J, Suppe J (2018). Proto-South China sea plate tectonics using subducted slab constraints from tomography. J. Earth Science.

[44] Hillier JK, Watts AB (2005). Relationship between depth and age in the North Pacific Ocean. J. Geophys. Res..

[45] Bechtel TD, Forsyth DW, Sharpton VL, Grieve RAF (1990). Variations in effective elastic thickness of the North American lithosphere. Nature.

[46] Poudjom Djomani YH, Fairhead JD, Griffin WL (1999). The flexural rigidity of Fennoscandia: Reflection of the tectonothermal age of the lithospheric mantle. Earth Planet. Sci. Lett..

[47] Crough ST (1983). Hotspot swells. Ann. Rev. Earth Planet. Sci..

[48] Li X, Kind R, Yuan X, Wolbern I, Hanka W (2004). Rejuvenation of the lithosphere by the Hawaiian plume. Nature.

[49] Laske G, Markee A, Orcutt JA, Wolfe CJ, Collins JA, Solomon SC, Detrick RS, Bercovici D, Hauri EH (2011). Asymmetric shallow mantle structure beneath the Hawaiian swell–evidence from Rayleigh waves recorded by the PLUME network. Geophys. J..

[50] Hickey-Vargas R, Yogodzinski GM, Ishizuka O, McCarthy A, Bizimis M, Kusano Y, Savov IP, Arculus R (2018). Origin of depleted basalts during subduction initiation and early development of the Izu-Bonin-Mariana island arc: Evidence from IODP expedition 351 site U1438. Amami-Sankaku Basin Geochimica et Cosmochimica Acta.

[51] Abers GA, van Keken PE, Kneller EA, Ferris A, Stachnik JC (2006). The thermal structure of subduction zones constrained by seismic imaging: Implications for slab dehydration and wedge flow. Earth Planet. Sci. Lett..

[52] Lin SC, Kuo BY, Chiao LY, van Keken PE (2005). Thermal plume models and melt generation in East Africa: A dynamic modeling approach. Earth Planet. Sci. Lett..

[53] Wessel P, Smith WHF (1998). New, improved version of generic mapping tools released. Eos Trans. AGU.

