# Supplementary material for: Thermal ages of the Huatung Basin determined from seismic waveform modeling: insights into Southeast Asia’s evolution
Source: Sci Rep. 2023 Sep 14;13:15201. doi: 10.1038/s41598-023-42454-x (PMC10502009; doi:10.1038/s41598-023-42454-x)
Supplement: Supplementary file 1 — Supplementary Information. [file 41598_2023_42454_MOESM1_ESM.docx]

Supporting Information for

**Thermal Ages of the Huatung Basin Determined from Seismic Waveform Modeling: Insights into Southeast Asia's Evolution**

Justin Yen-Ting Ko^1*^, Ban-Yuan Kuo^2^, Shu-Chuan Lin^2^, and Yu-Sheng Hung^1^

^1^ Institute of Oceanography, National Taiwan University, Taipei, Taiwan

^2^ Institute of Earth Sciences, Academia Sinica, Taipei, Taiwan

*Corresponding author: Justin Yen-Ting Ko ([justinko@ntu.edu.tw](mailto:justinko@ntu.edu.tw))

Contents of this file

1. Uncertainty

Extended Data Table 1-2

Extended Data Figure 1-21

1. Uncertainty
   1. Three-Dimensional Effects

We proceeded under the assumption that the intricacies of waveform behaviors can be primarily accounted for by two-dimensional (2D) models. However, it is essential to acknowledge that three-dimensional (3D) effects must also be taken into account to gain a more comprehensive understanding of the slab's morphology. In order to assess whether lateral variations in P-wave velocity contribute to our simulation outcomes, we established a model database encompassing three distinct events, each spanning approximately 25° in azimuth. Subsequently, a grid search was conducted for each event. Notably, we observed variations in the resolved parameters among the different events (Extended Data Table 2), yet these deviations were confined to a relatively narrow range. It is noteworthy that utilizing the optimal model derived from AI2007 yielded a reasonable fit to the amplitude patterns of the other events as well (Extended Data Figure 10). This observation suggests that the most prominent deviations in amplitude measurements could be predominantly influenced by the velocity anomalies in the direction perpendicular to the trench. Our investigation into the lateral variations in velocity structures indicated a relatively minor impact within our study area.

Owing to the inherent trade-offs between strike and dip values, accurately determining the true dip of the slab using a single 2D section is challenging. This complexity can result in potential errors that manifest in our resolving parameters. By considering three events with azimuthal differences of approximately 25°, we aimed to alleviate this issue. This approach is particularly beneficial in addressing the uncertainties associated with the determination of slab dip. The accurate identification of double arrivals exhibited a strong correlation with the alignment between the edges of the slab and the ray paths of the seismic events. In this context, the waveforms stemming from the 2007 Aleutian Islands event revealed a more pronounced multipath effect. This observation implies that the dip angle of the slab ideally should match the incident angle of the propagating wavefields to achieve optimal results.

- 1. Single dip angle

Uncertainty may arise from the assumption with a single dip angle corresponding to our idealized slab model. The oblique subduction of the Philippine Sea Plate (PSP) creates a subduction–collision complex in offshore Taiwan. The relocated locations reveal the folding of the slab, which leads to a change in dip directions (Chou et al., 2006). The assumption with multiple dips may delineate the morphology of the slab more accurately. However, the determination of the bending slab model requires a considerably greater number of parameters, vastly expanding the model space and resulting in substantial increases in the simulation time. Even with a simplified slab geometry, accuracy in estimating the thermal age corresponding to the first-order feature of the slab morphology should be maintained. Giving the slab a bending parameter would be more realistic but would not result in a notable difference in the age estimate.

1.3 Local Structures

Earthquakes with different azimuths may have similar patterns of amplitude and waveforms if local structural anomalies are strong. We measured the amplitude fluctuations from other directions to examine whether local structures dominated the abnormal patterns. Figures S16 display the amplitude anomalies recorded by the local networks for earthquakes in Tonga and the Hindu Kush. No clear patterns in amplitude measurements were observable, indicating that local anomalies contribute little to the focusing–defocusing patterns observed. Moreover, according to the BB′ velocity profile established by Huang et al. (2014), the evidence for a subducting Eurasian Plate (EP) in the southern portion of Taiwan is clear. Compared with the subducting PSP plate, the EP does not have a precise dip angle but exists as a high P wave velocity portion in situ. Therefore, the methodology of this study is more applicable to an investigation of the subducting PSP plate than to an investigation of the EA plate.

| ­­DATE | Time | Longitude (°) | Latitude (°) | Depth(km) | Mw |
| --- | --- | --- | --- | --- | --- |
| **2007/04/29(N)** | **12:41:58** | **-179.96** | **52.01** | **126.9** | **6.2** |
| 2007/05/30(N) | 20:22:13 | 157.22 | 52.15 | 122.3 | 6.4 |
| 2007/09/03(N) | 16:14:54 | 150.1 | 45.8 | 100.6 | 6.2 |
| **2013/10/01(N)** | **03:38:21** | **152.9** | **53.14** | **578.4** | **6.7** |
| 2017/10/08(N) | 22:34:33 | 176.8 | 52.45 | 111.8 | 6.6 |
| 2018/10/13(N) | 11:10:22 | 153.24 | 52.85 | 486.81 | 6.3 |
| 2019/11/20(N) | 08:26:07 | 153.69 | 53.16 | 486.81 | 6.3 |
| **2020/02/13(N)** | **10:33:44** | **148.93** | **45.63** | **144** | **7.0** |
| 2020/09/15(N) | 03:41:28 | 158.42 | 55.97 | 344 | 6.4 |
| 2015/01/23(E) | 03:47:27 | 168.55 | -17.1 | 223.9 | 6.8 |
| 2017/02/24(E) | 17:28:44 | -178.8 | -23.26 | 414.9 | 6.9 |
| 2017/05/09(E) | 13:52:10 | 167.37 | -14.59 | 169 | 6.8 |
| 2018/09/30(E) | 10:52:24 | -178.08 | -18.35 | 564.1 | 6.7 |
| 2018/11/18(E) | 20:25:46 | -178.92 | -17.87 | 540 | 6.8 |
| 2019/07/13(E) | 15:02:33 | 168 | -16.2 | 181 | 6.6 |
| 2019/09/01(E) | 15:54:20 | -178.57 | -20.36 | 591 | 6.6 |
| 2016/04/10(W) | 10:28:58 | 71.13 | 36.47 | 212 | 6.6 |
| 2018/05/09(W) | 10:41:45 | 71.38 | 36.99 | 116 | 6.2 |

**Extended Data Table 1 | Earthquakes included in this study.** Three seismic events with high-quality data, namely the Aleutian Islands event in 2007 (AI2007), the Kamchatka Peninsula event in 2013 (KP2013), and the Northeastern Japan event in 2020 (NJ2020), have been identified and marked in bold. The locations of the events are indicated relative to Taiwan using the abbreviations (N), (E), and (W) for north, east, and west, respectively.

| Model parameters | AI2007 | KP2013 | NJ2020 |
| --- | --- | --- | --- |
| Velocity perturbation (dV) | 6 % | 5.5% | 6% |
| Slab length (L) | 400 km | 450 km | 400 km |
| Slab width (W) | 150 km | 150 km | 125 km |
| Slab dipping angle (θ) | 55° | 55° | 60° |
| Slab sharpness (SP) | 0.133 1/s | 0.11 1/s | 0.12 1/s |

**Extended Data Table 2 | Optimal slab model parameters for AI2007, KP2013 and NJ2020.**


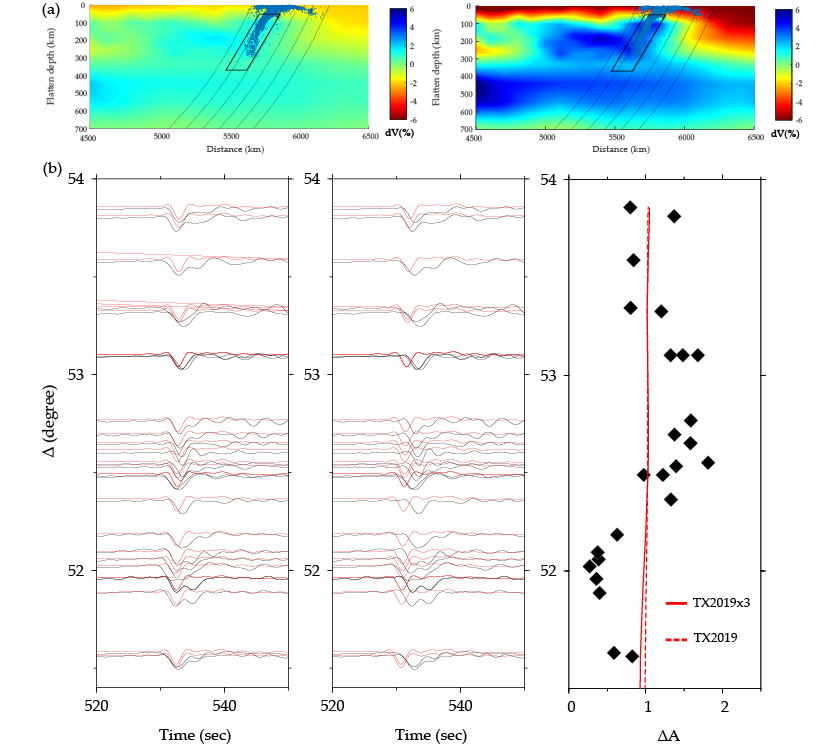


**Extended Data Figure 1 | Evaluation of TX2019 Models and 3-Times Inflated TX2019. a,** Illustration of the TX2019 model (left) compared with the TX2019 model incorporating 3-times inflation of velocity anomalies (TX2019x3, right). **b,** Detailed comparison of waveform patterns between the TX2019 model (left) and the TX2019x3 model (middle), along with corresponding amplitude patterns (right). The red traces depict synthetics, while the black traces represent the observed data.


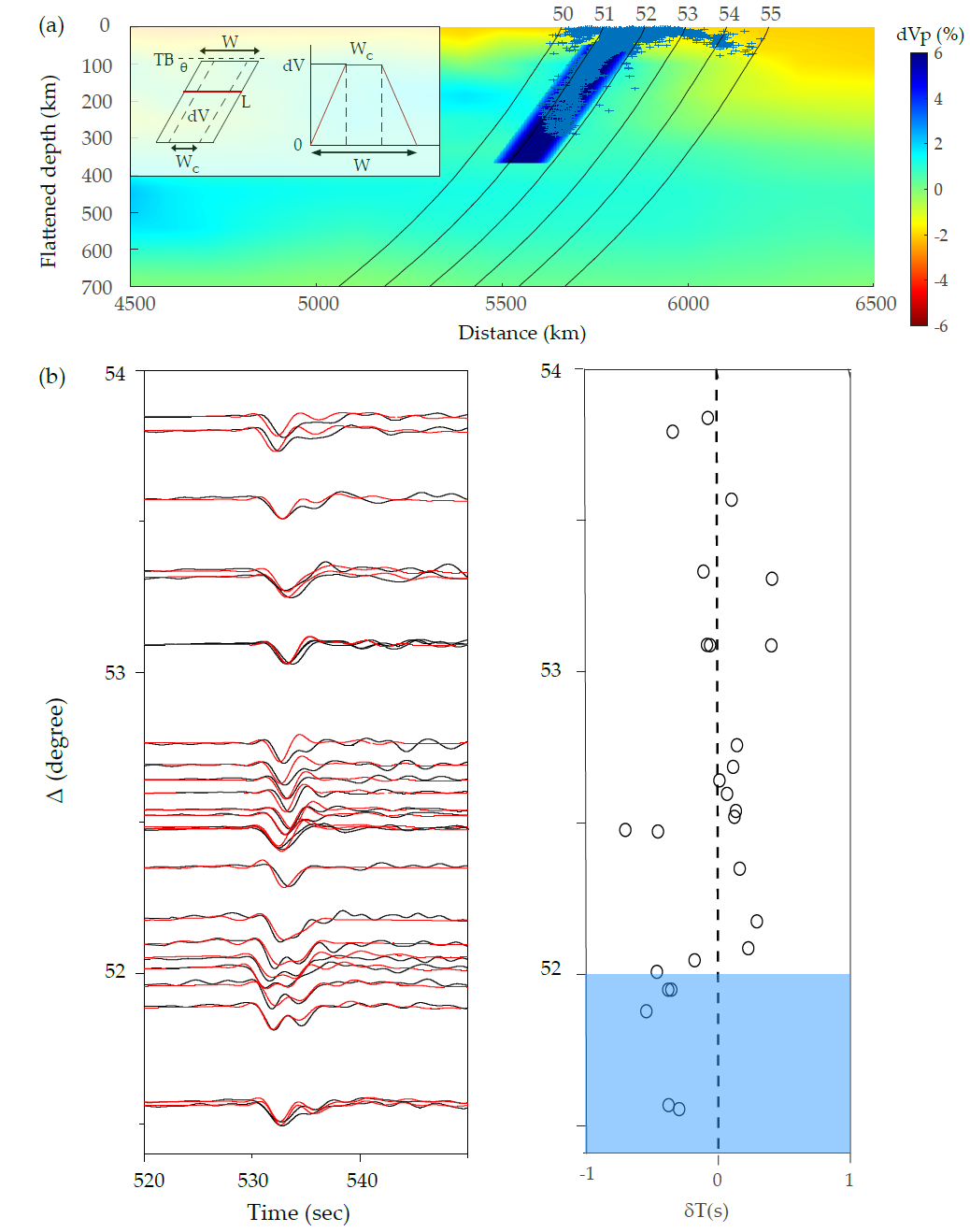


**Extended Data Figure 2 | Travel-time anomalies and waveform fitting of the optimal slab parameter for AI2007. a,** Optimal slab model embedded into TX2019 slab, with ray paths representing different epicentral distances. Blue dots represent the background seismicity. The inset on the left presents the model parameters used to characterize the idealized slab, including top boundary (TB), slab dip ($\theta$), slab width (W), width of the slab core (W_C_), and velocity perturbation within the slab core ($\delta V$). The inset on the right shows the velocity structures within the idealized slab model. **b,** Fitting results for waveforms (left) and the observations of travel-time anomalies (right).


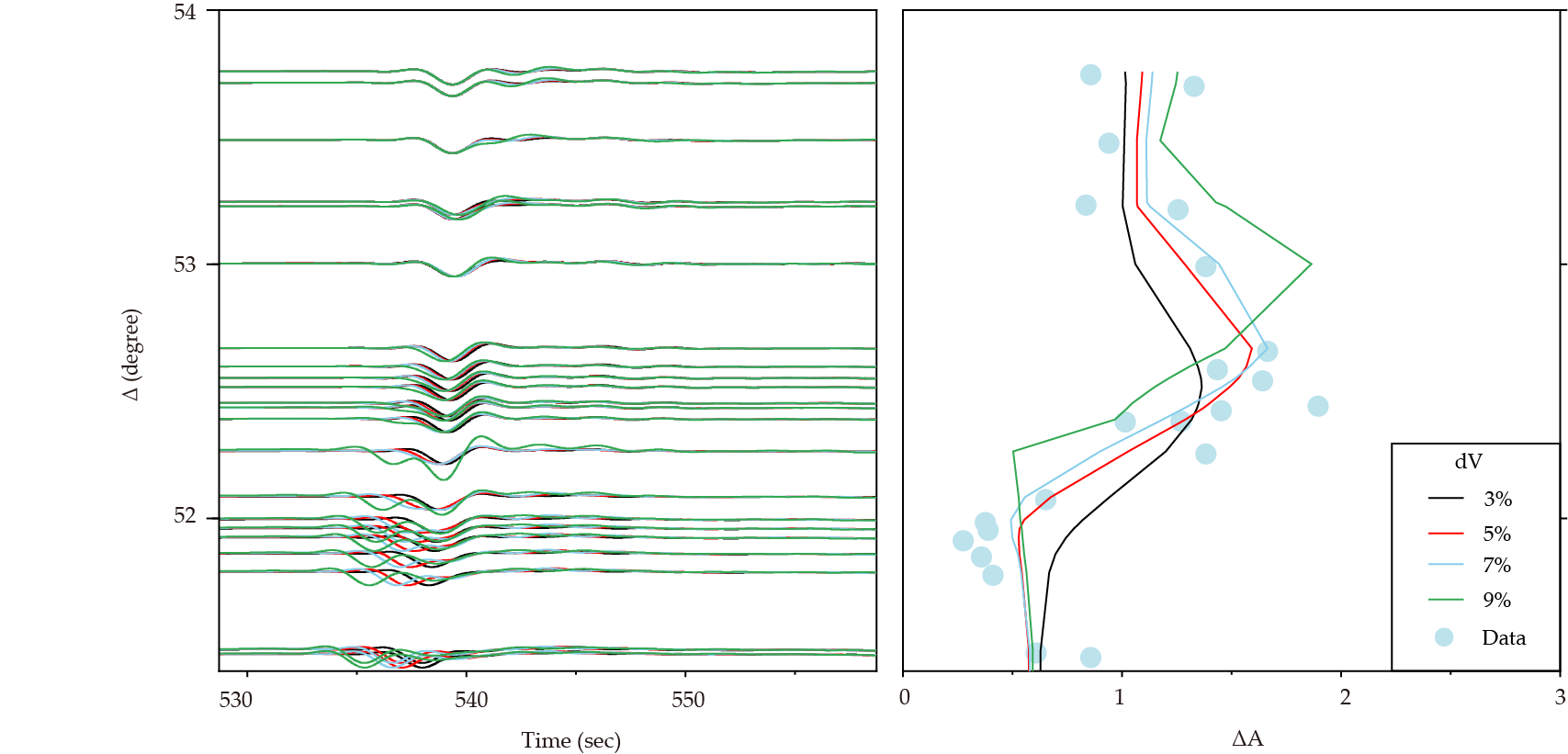


**Extended Data Figure 3 | Sensitivity testing of the velocity perturbation within the slab.** The blue dots represent the data from the 2007 Aleutian Islands event (AI2007). Different colors indicate the different velocity perturbations within the slab core.


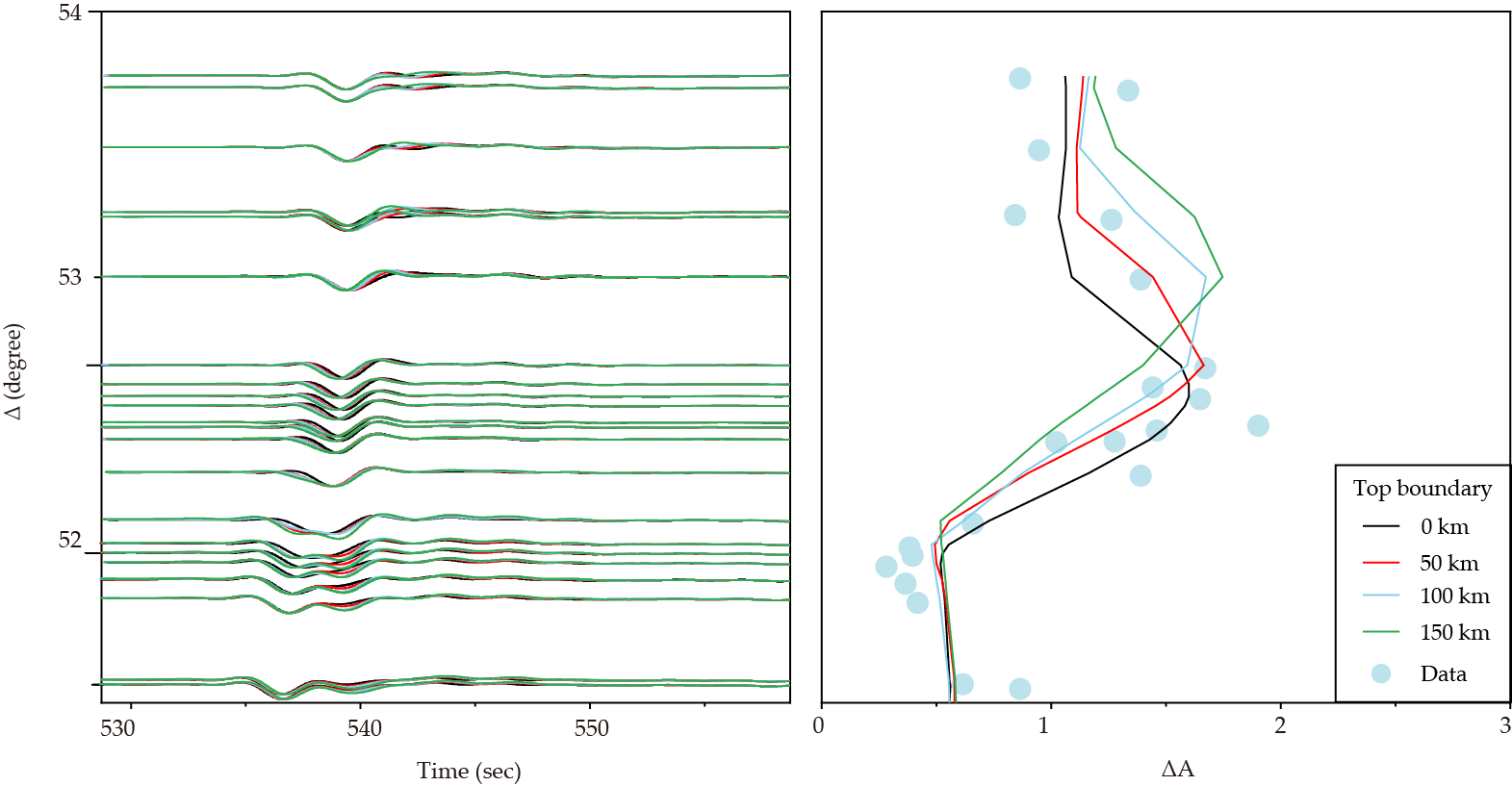


**Extended Data Figure 4 | Sensitivity testing of the top boundary of the slab.** The blue dots are data from AI2007. Different colors indicate the different depths of the top boundaries.


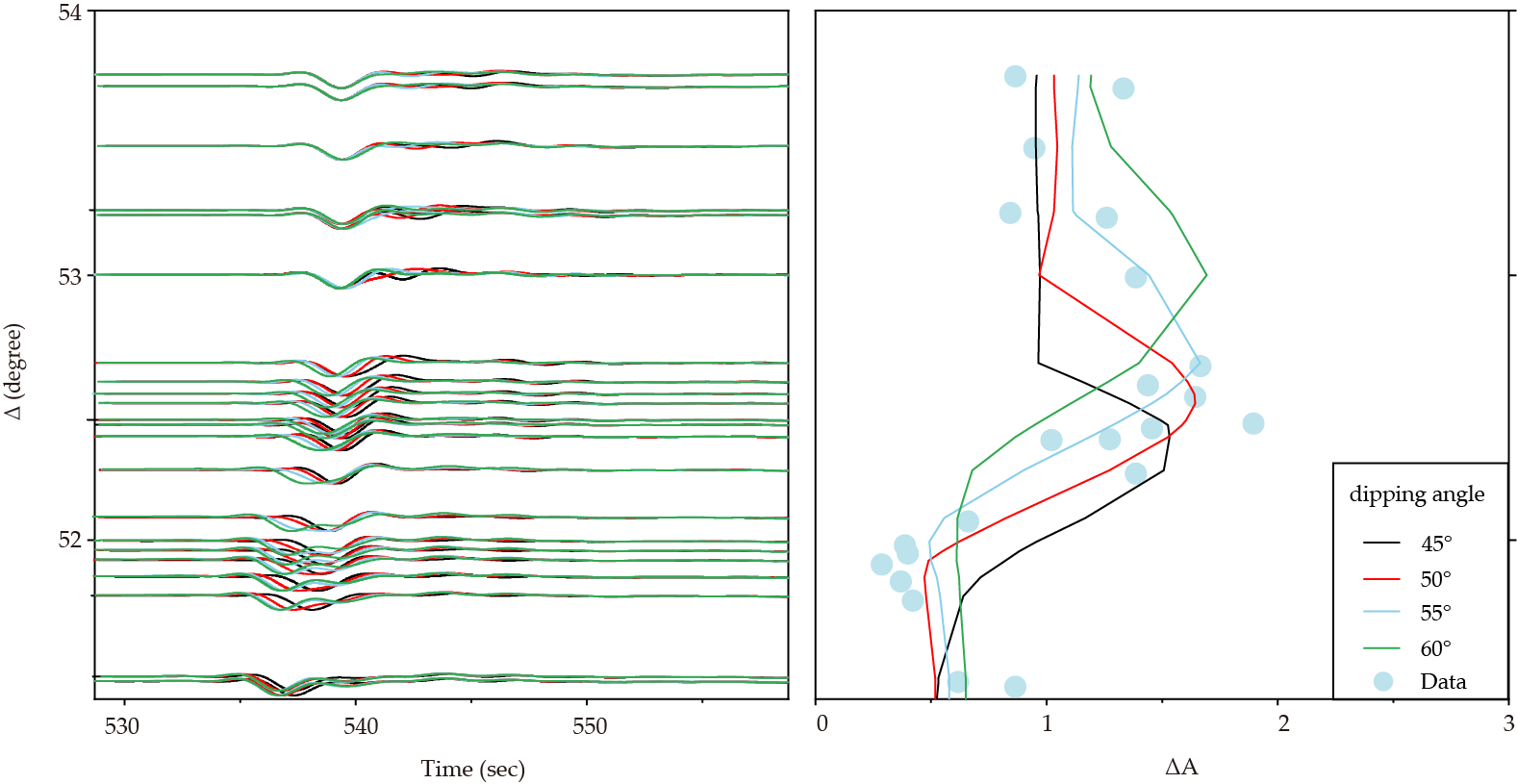


**Extended Data Figure 5 | Sensitivity testing of the dip angle of the slab.** The blue dots are data from AI2007. Different colors indicate the different dip angles of the slab.


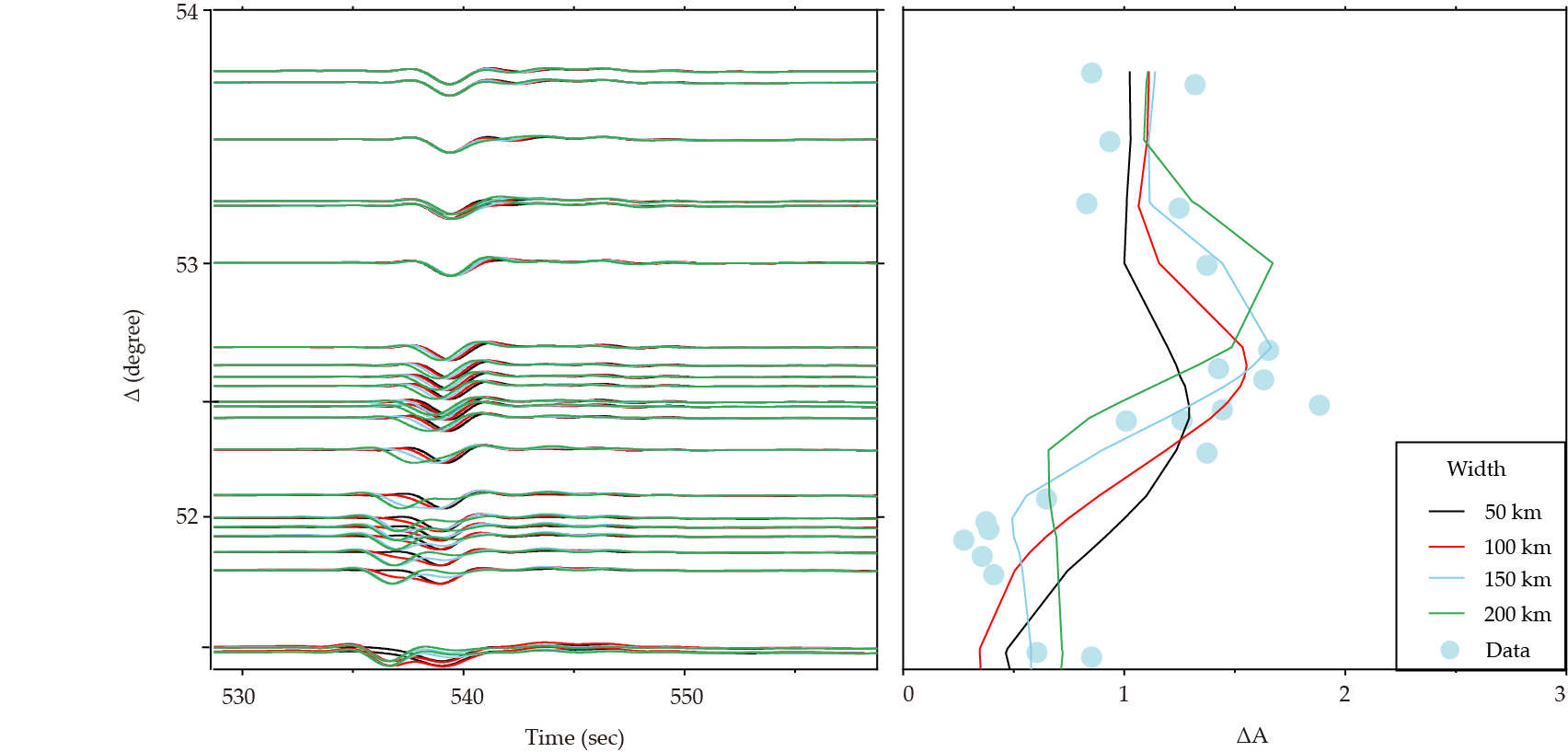


**Extended Data Figure 6 | Sensitivity testing of the width of the slab.** The blue dots are data from AI2007. Different colors indicate the different widths of the slab.


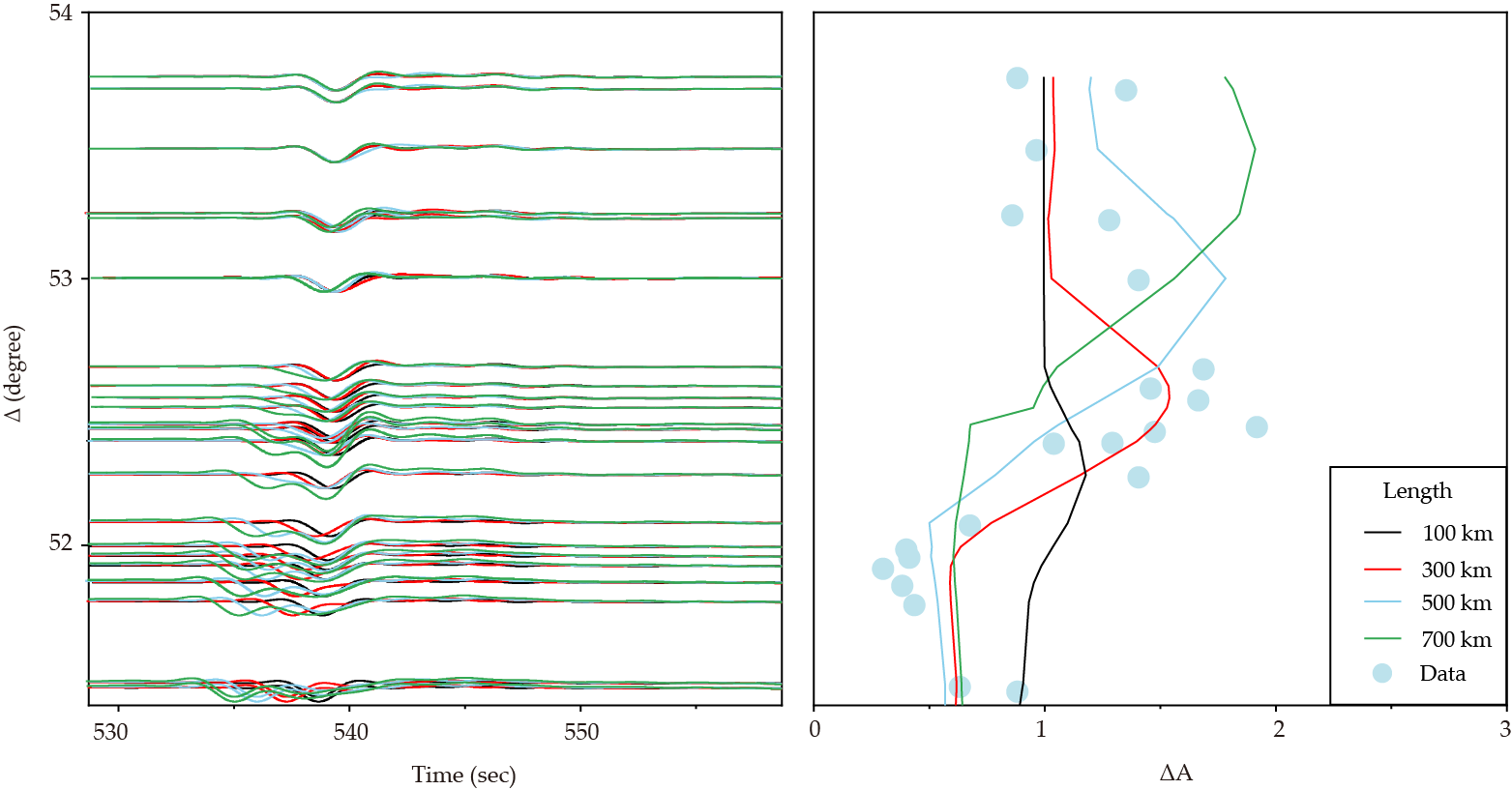


**Extended Data Figure 7 | Sensitivity testing of the length of the slab.** Sensitivity testing of the length of the slab. The blue dots are data from AI2007. Different colors indicate the different lengths of the slab.


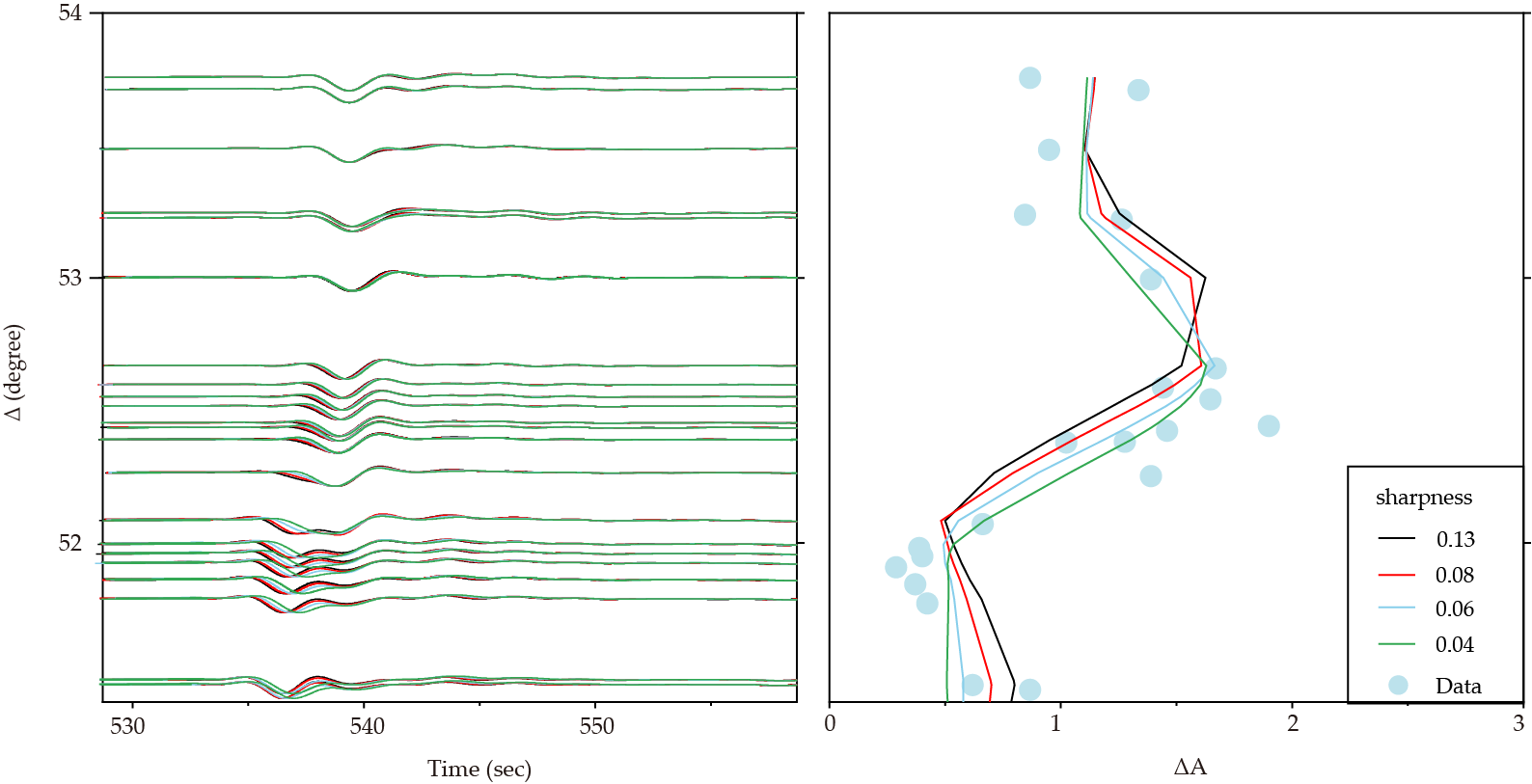


**Extended Data Figure 8 | Sensitivity testing of the sharpness of the slab edge.** Sensitivity testing of the sharpness of the slab edge. The blue dots are data from AI2007. Different colors indicate the different sharpness values of the slab edge.


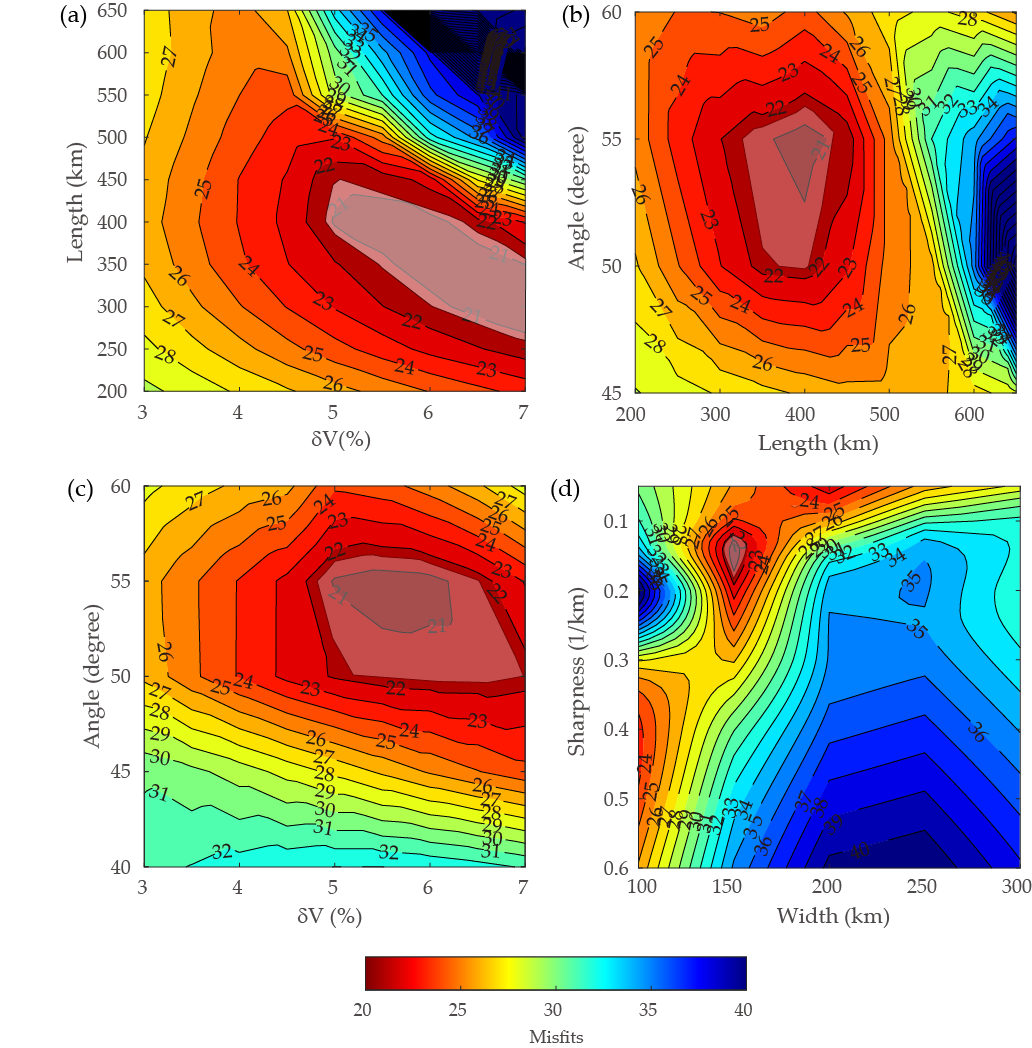


**Extended Data Figure 9 | Misfit surfaces with contours demonstrating the values of the cost function with different model variables**: **a,** $\delta V$ and length, **b**, length and dip angle, **c**, $\delta V$ and dip angle, and **d,** width and slab edge sharpness. The shaded area indicates the regions in which the misfit deviates from the global minimum by less than 5%.


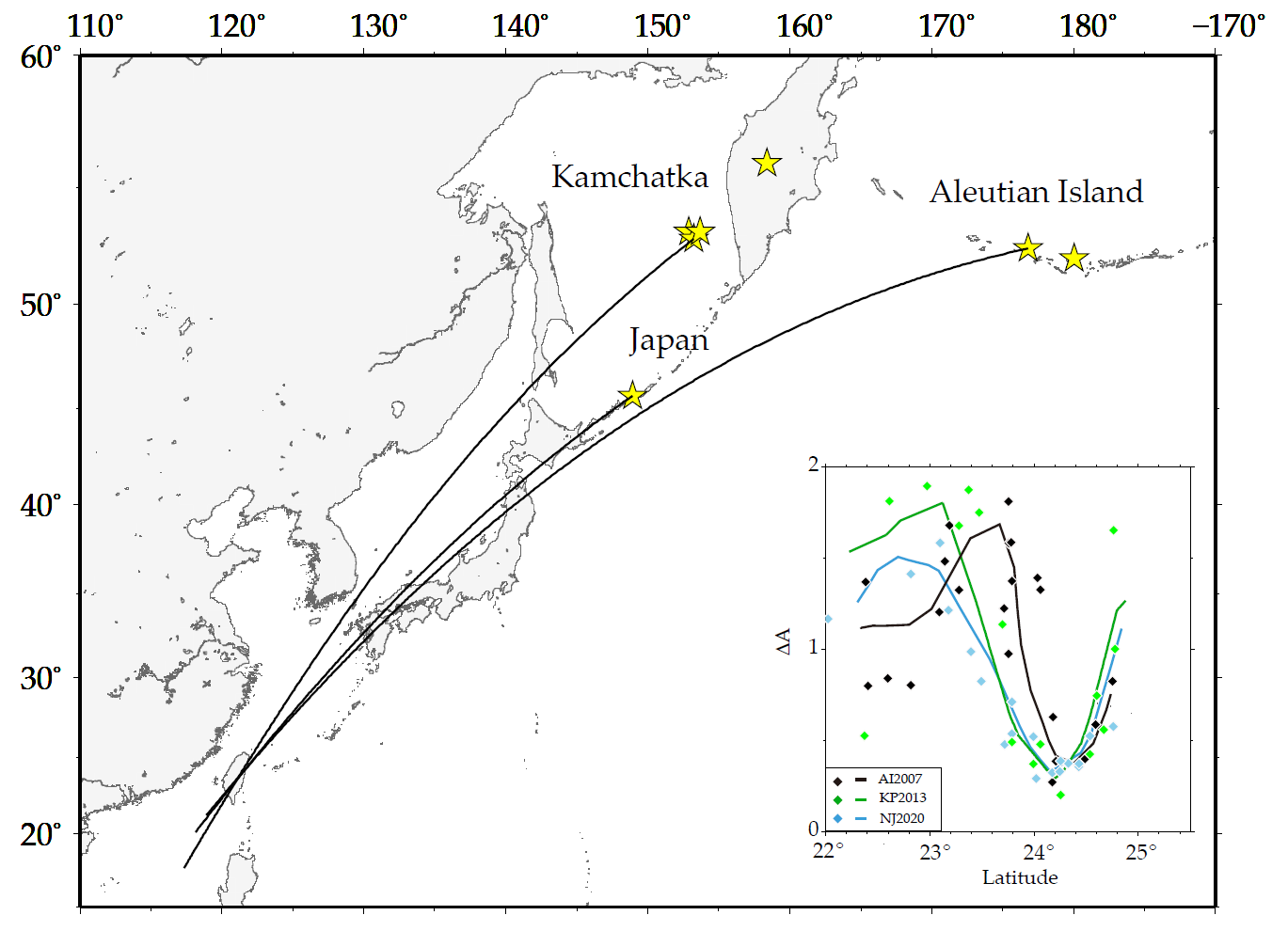


**Extended Data Figure 10 | Map view of events used in the simulations (yellow stars) and the amplitude anomalies (inset).** Map view of events used in the simulations (yellow stars) and the amplitude anomalies (inset). The inset demonstrates that the amplitude patterns for different events (colored dots) were similar. Embedding optimal slab models into different 2D velocity structures (obtained from TX2019slab with different azimuths) yielded a favorable fit with the data.


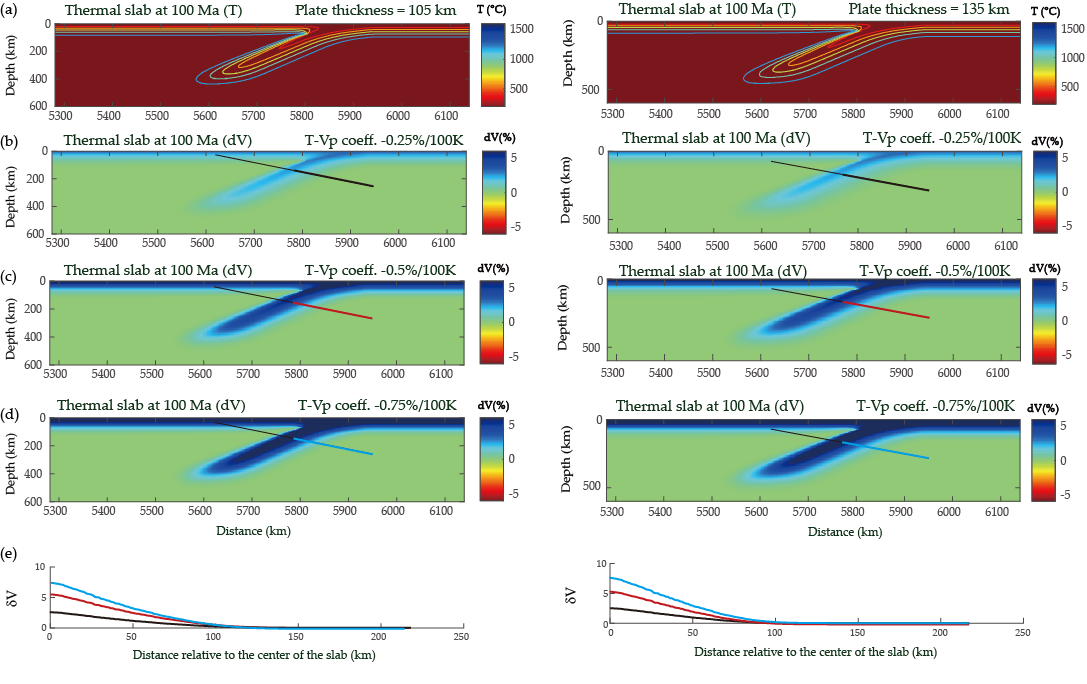


**Extended Data Figure 11 |** **Thermal slab model at 100 Ma for 105-km and 135-km plate models.** **a,** Isothermal contour plot depicting the thermal slab model with a plate thickness of 105 km (left) and 135 km (right). **b,** Velocity perturbation of the thermal slab with a plate thickness of 105 km (left) and 135 km (right), derived from the thermal-velocity relationship of -0.25%/100K. **c,** Velocity perturbation of the thermal slab with a plate thickness of 105 km (left) and 135 km (right), derived from the thermal-velocity relationship of -0.5%/100K. **d,** Velocity perturbation of the thermal slab with a plate thickness of 105 km (left) and 135 km (right), derived from the thermal-velocity relationship of -0.75%/100K. **e,** Velocity gradient profiles extracted from the color-coded transection across each thermal slab.


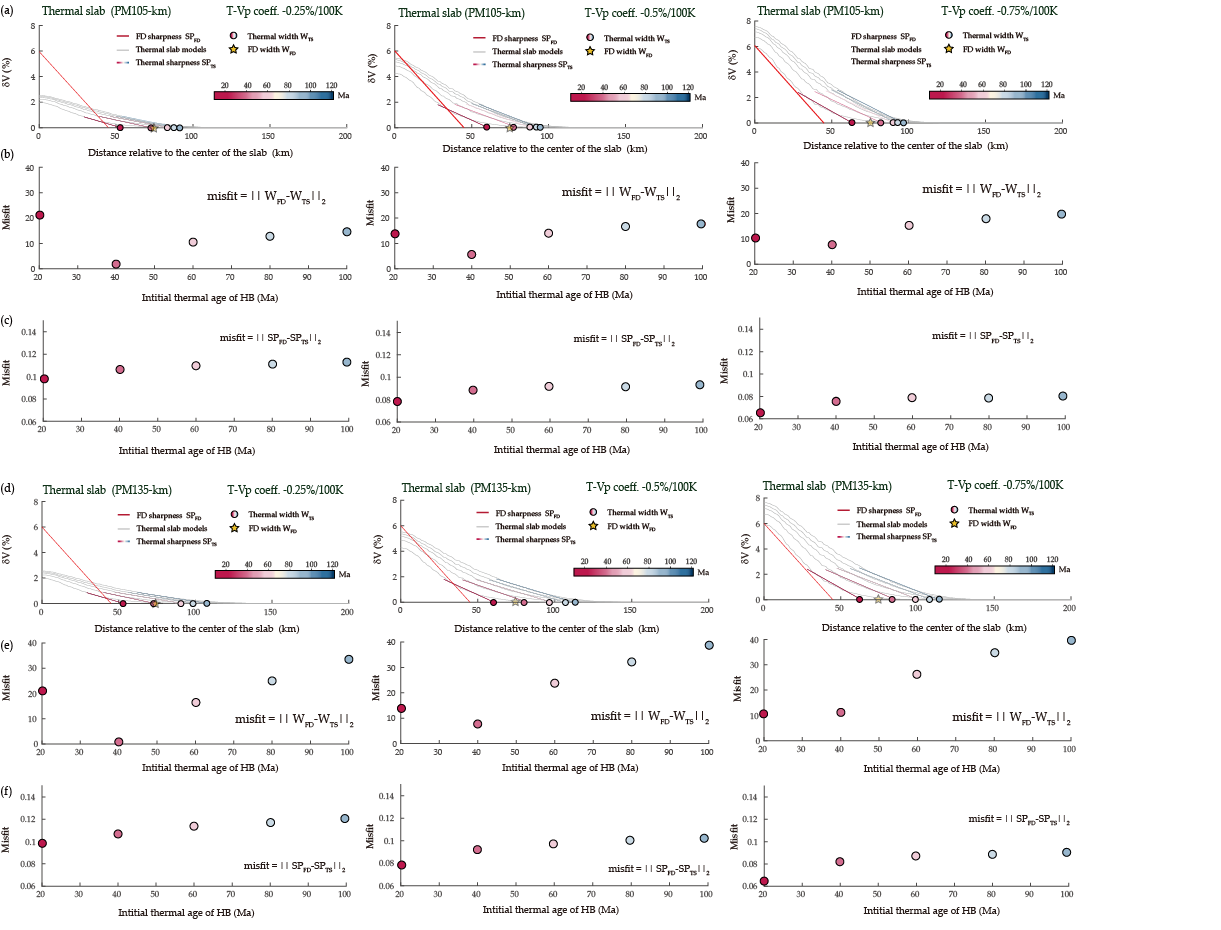


**Extended Data Figure 12 |** **Ages determination of the thermal slab models for 105-km and 135-km plate models.** **a,** The thermal slab models for 105-km plate models yielded $\delta V$ values, derived from the thermal-velocity relationship of -0.25%/100K (left), -0.5%/100K (left), and -0.75%/100K (right), with the gray lines indicating the predictions obtained from age-dependent models. The left-to-right sequence of initial thermal ages for HB are 20, 40, 60, 80, 100 Ma, respectively. The color-coded lines indicate the linear regression results of $\delta V$ values ranging from the edge of the thermal slab core to 0.1. The yellow star and color-coded dots indicate the estimated width of the slab from seismic and thermal slab models, respectively. **b,** Misfit of slab width between the seismic and thermal slab models at different ages (color coded). **c,** Misfit of sharpness between the seismic and thermal slab models at different ages (color coded). **d,** The thermal slab models for 135-km plate models yielded $\delta V$ values, derived from the thermal-velocity relationship of -0.25%/100K (left), -0.5%/100K (left), and -0.75%/100K (right), with the gray lines indicating the predictions obtained from age-dependent models. The left-to-right sequence of initial thermal ages for HB are 20, 40, 60, 80, 100 Ma, respectively. The color-coded lines indicate the linear regression results of $\delta V$ values ranging from the edge of the thermal slab core to 0.1. The yellow star and color-coded dots indicate the estimated width of the slab from seismic and thermal slab models, respectively. **e,** Misfit of slab width between the seismic and thermal slab models at different ages (color coded). **f,** Misfit of sharpness between the seismic and thermal slab models at different ages (color coded).


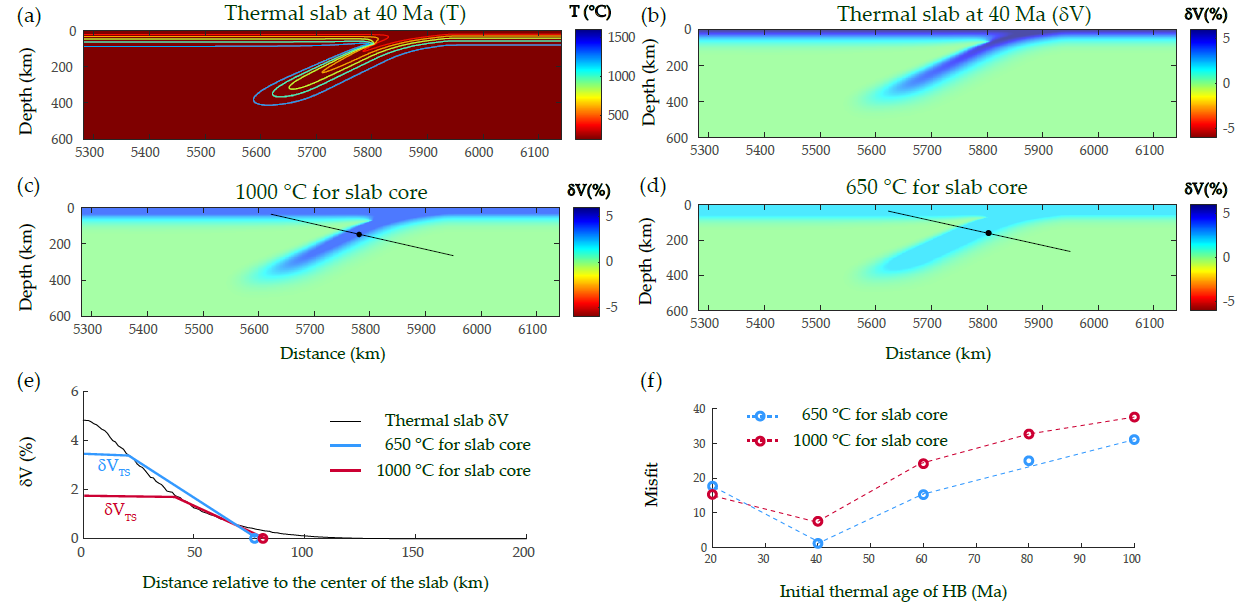


**Extended Data Figure 13 | Thermal slab model at 40 Ma and its age estimations.** **a,** Isothermal contour plot illustrating the thermal slab model with a plate thickness of 135 km. **b,** Velocity perturbation of the thermal slab, obtained through conversion from the thermal-velocity relationship of -0.5%/100K. **c,** Velocity perturbation of the thermal slab, featuring a uniform velocity within the slab core where the temperature is less than 1000°C. **d,** Velocity perturbation of the thermal slab, showcasing a consistent velocity within the slab core where the temperature is less than 650°C. **e,** Idealized velocity structures derived from the thermal slab model, depicted by red and blue curves. The half width of the thermal slab is determined by fitting a linear slope to the velocity decline from the core boundary to 0.1 and extrapolating it to zero on the x-axis. **f,** Comparison of the misfit in slab width between the seismic and thermal slab models at different ages, color-coded to represent varying assumed temperature of the slab core for the 135-km thick plate model.


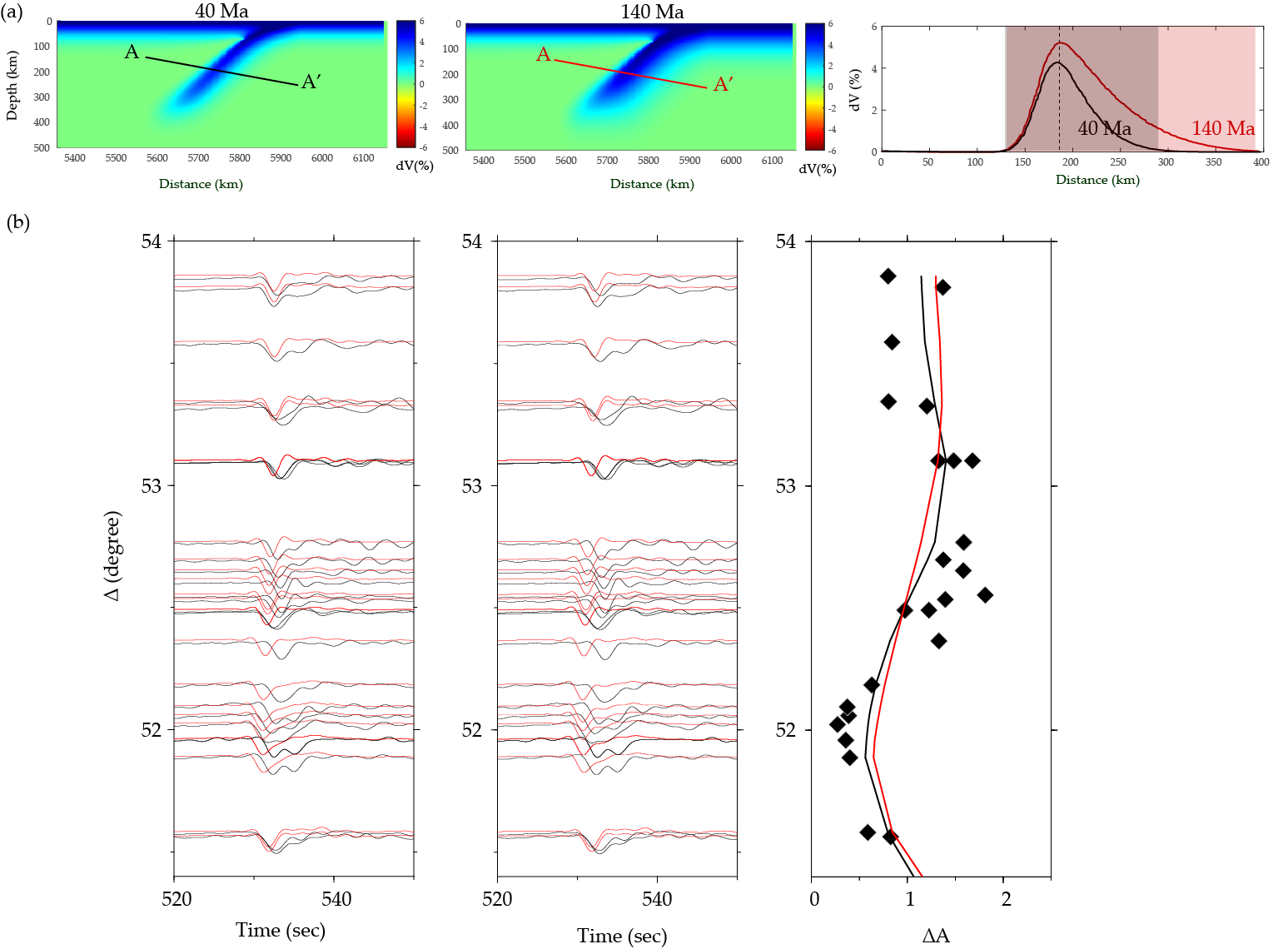


**Extended Data Figure 14 |** **Numerical assessments investigating slab models at ages 40 Ma and 140 Ma.** **a,** Illustration of the thermal slab models corresponding to 40 Ma (left) and 140 Ma (middle), accompanied by the extracted velocity gradient profiles observed across the AA’ transection of each thermal slab. The thermal slab's thickness is assessed by measuring the distance between the top and bottom boundaries where δV=0, as indicated by varying shaded colors. **b,** An extensive juxtaposition of waveform patterns encompassing the models at 40 Ma (left) and 140 Ma (middle), alongside the associated amplitude patterns (right). In the amplitude plot, the black diamond signifies the data points, while the red and black lines depict the amplitude patterns at 40 Ma and 140 Ma, respectively.


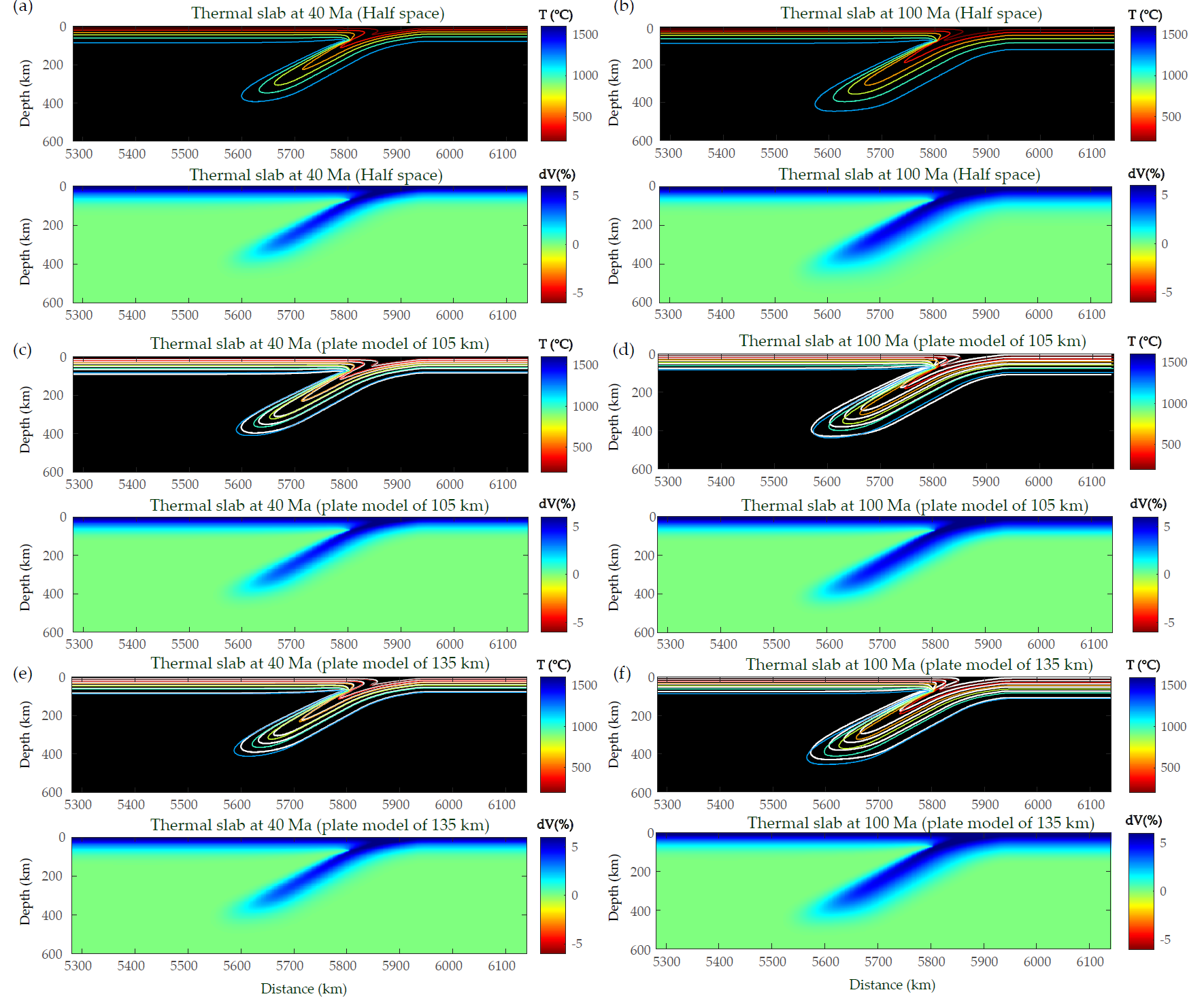


**Extended Data Figure 15 | Comparative Analysis of Thermal Models: Half-Space vs. Plate Model at 40Ma and 100Ma. a,** Isothermal contour plot illustrating the thermal slab at 40 Ma utilizing the half-space assumption (top) and the corresponding velocity perturbation of the thermal slab, derived through conversion from the thermal-velocity relationship of -0.5%/100K (bottom). **b,** Isothermal contour plot depicting the thermal slab at 100 Ma based on the half-space assumption (top) and the corresponding velocity perturbation of the thermal slab, derived through conversion from the thermal-velocity relationship of -0.5%/100K (bottom). **c,** Isothermal contour plot portraying the thermal slab at 40Ma employing the plate model with a thickness of 105 km (top) and the associated velocity perturbation of the thermal slab, obtained through conversion from the thermal-velocity relationship of -0.5%/100K (bottom). In the velocity plot, the white lines delineate the isothermal contour of the thermal slab using the half-space model. **d,** Isothermal contour plot illustrating the thermal slab at 100Ma using the plate model with a thickness of 105 km (top) and the corresponding velocity perturbation of the thermal slab, derived through conversion from the thermal-velocity relationship of -0.5%/100K (bottom). In the velocity plot, the white lines signify the isothermal contour of the thermal slab using the half-space model. **e,** Isothermal contour plot showcasing the thermal slab at 40Ma employing the plate model with a thickness of 135 km (top) and the associated velocity perturbation of the thermal slab, obtained through conversion from the thermal-velocity relationship of -0.5%/100K (bottom). In the velocity plot, the white lines indicate the isothermal contour of the thermal slab using the half-space model. **f,** Isothermal contour plot depicting the thermal slab at 100Ma using the plate model with a thickness of 135 km (top) and the corresponding velocity perturbation of the thermal slab, derived through conversion from the thermal-velocity relationship of -0.5%/100K (bottom). In the velocity plot, the white lines denote the isothermal contour of the thermal slab using the half-space model.


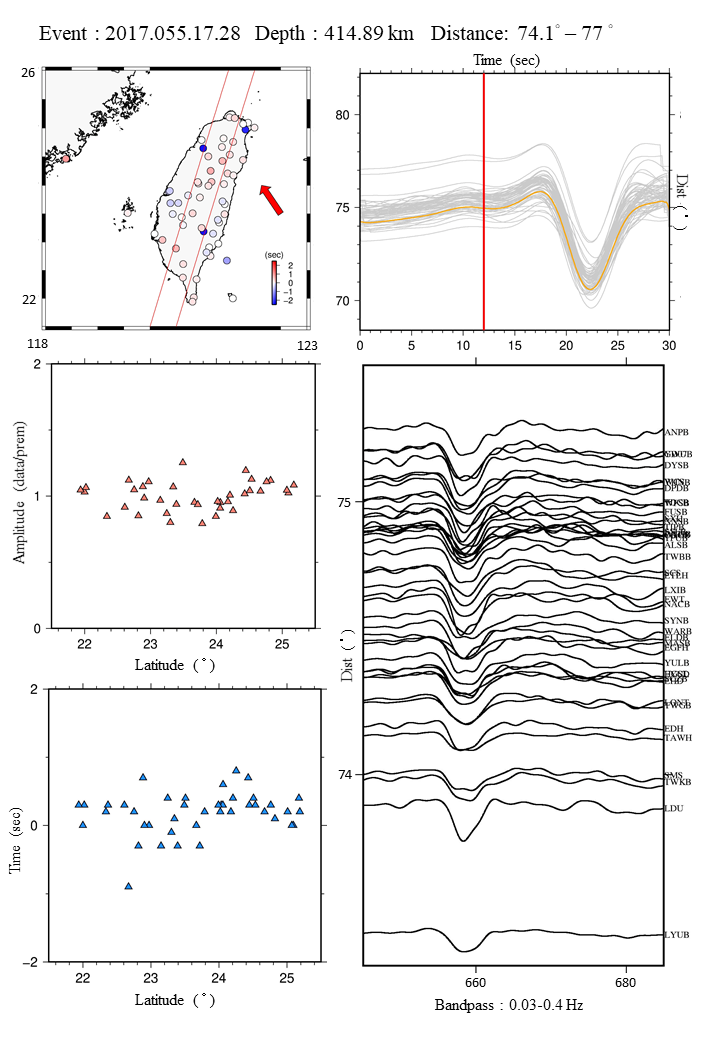

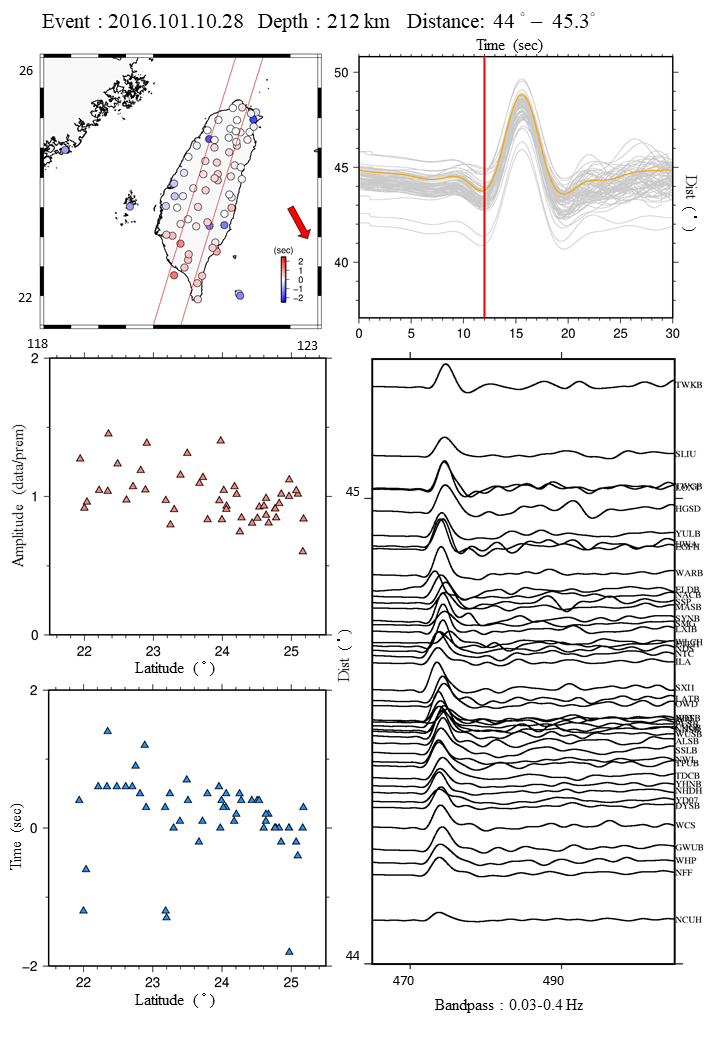


**Extended Data Figure 16 | Amplitude anomalies of earthquakes occurring in Tonga (left) and the Hindu** **Kush (right).**


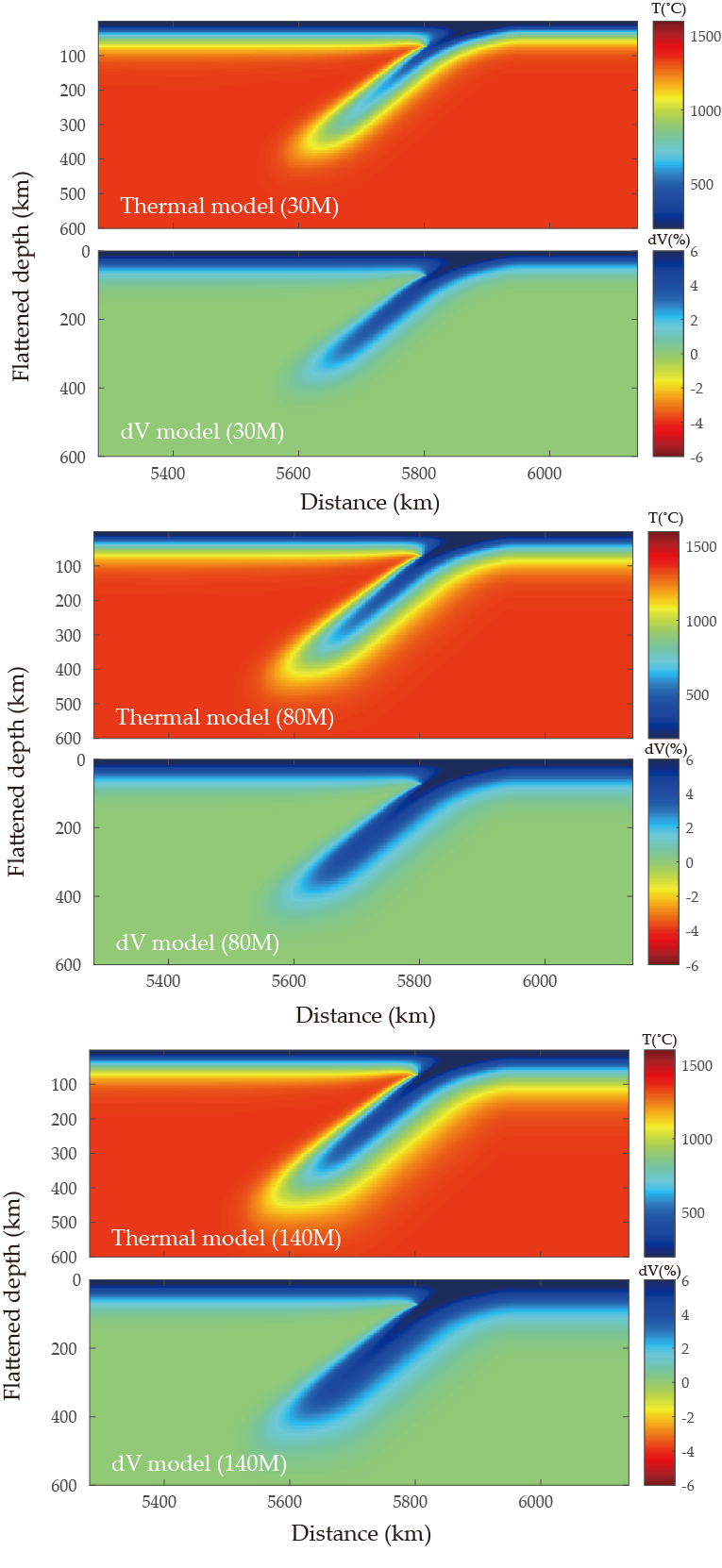
**­­­­**

**Extended Data Figure 17 | Thermal slab and converted velocity structures at 30 Ma (top), 80 Ma (middle), and 140 Ma (bottom).** Thermal slab and converted velocity structures at 30 Ma (top), 80 Ma (middle), and 140 Ma (bottom). The setup of the thermal models closely follows the stage-I models of Lin et al. (2010), using a widely-used approach for the thermal structure of the subduction zones, in which a dynamical mantle wedge is driven by a kinematic boundary of subduction (e.g., van Keken et al., 2008). Here a zero-temperature gradient is assumed at both side boundaries and the bottom, while the temperature is fixed at the top boundary.  A rigid overriding lithosphere of 25 km is prescribed, and the thickness of the rigid forearc lithosphere is 70 km. More details of the model parameters and the rheology can be found in Lin et al. (2010).


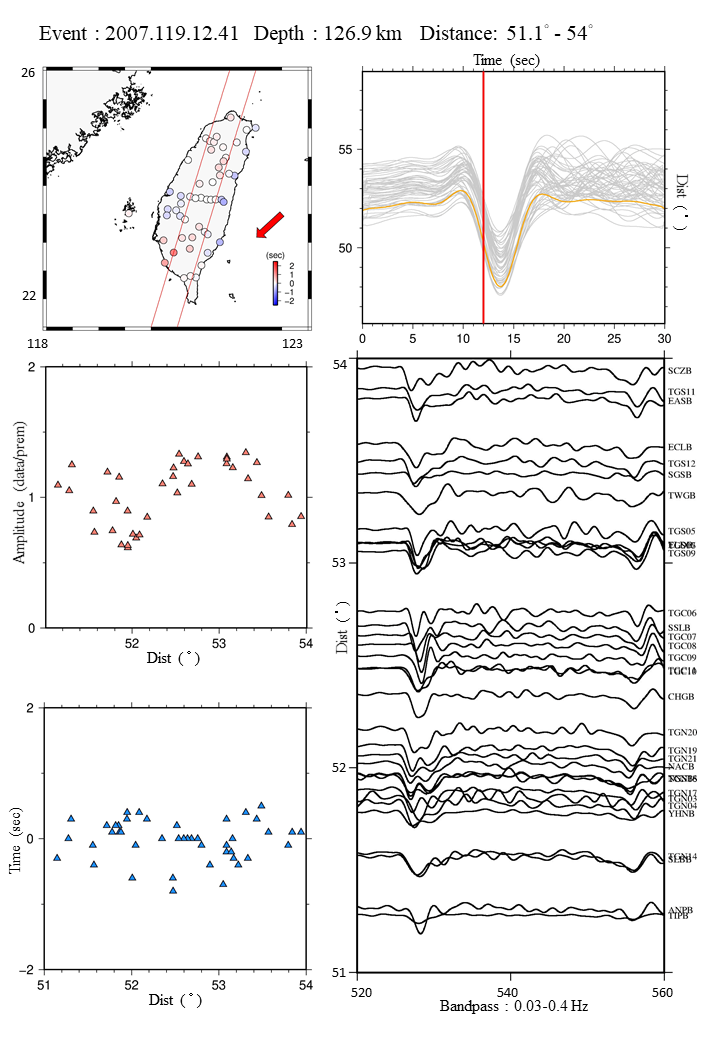

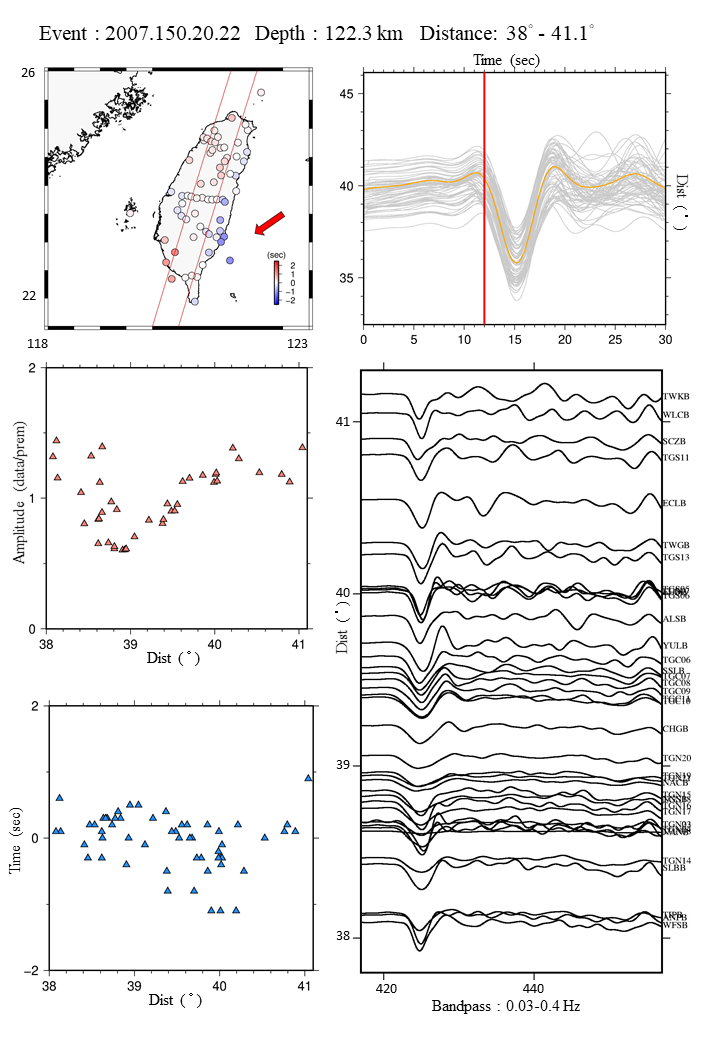

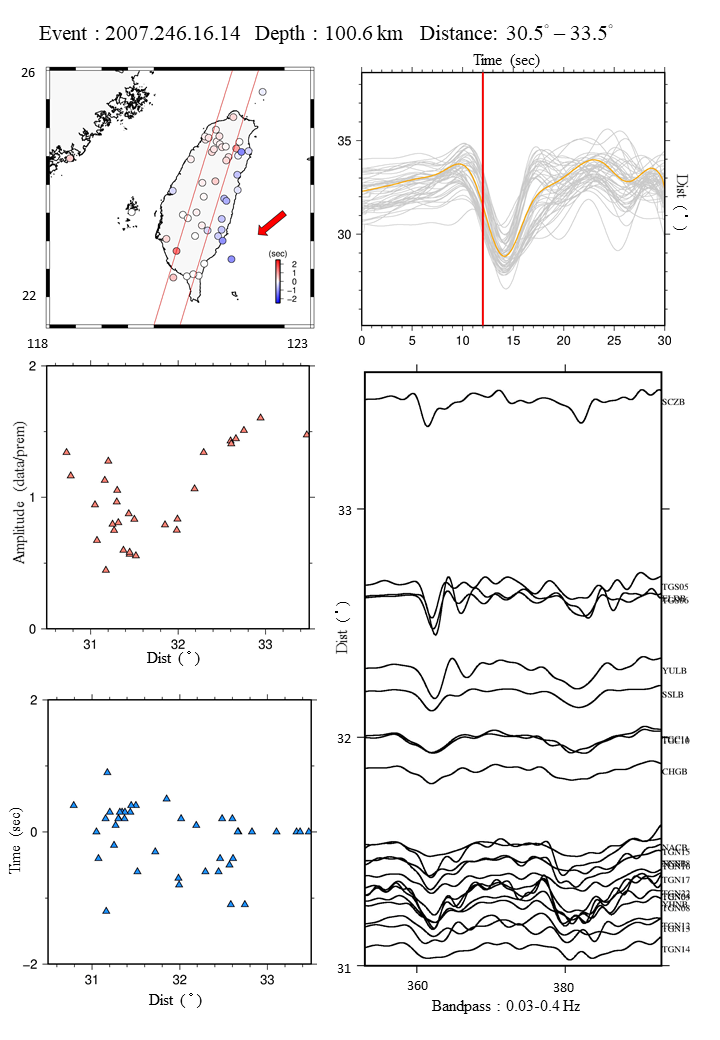


2007/05/30

2007/09/03

2007/04/29


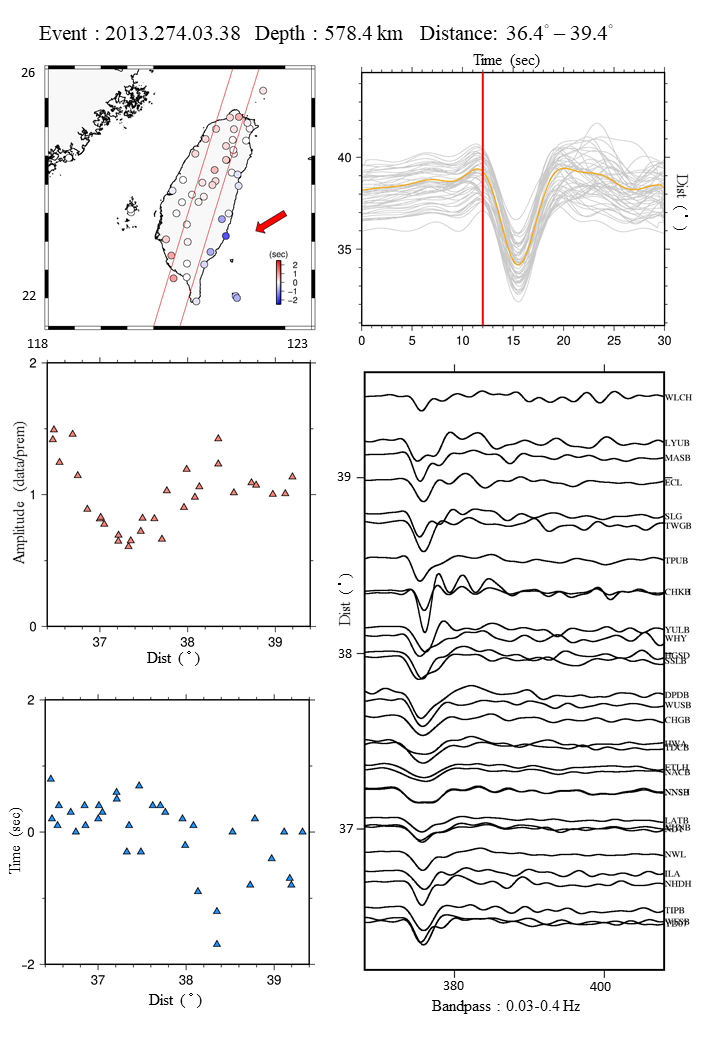

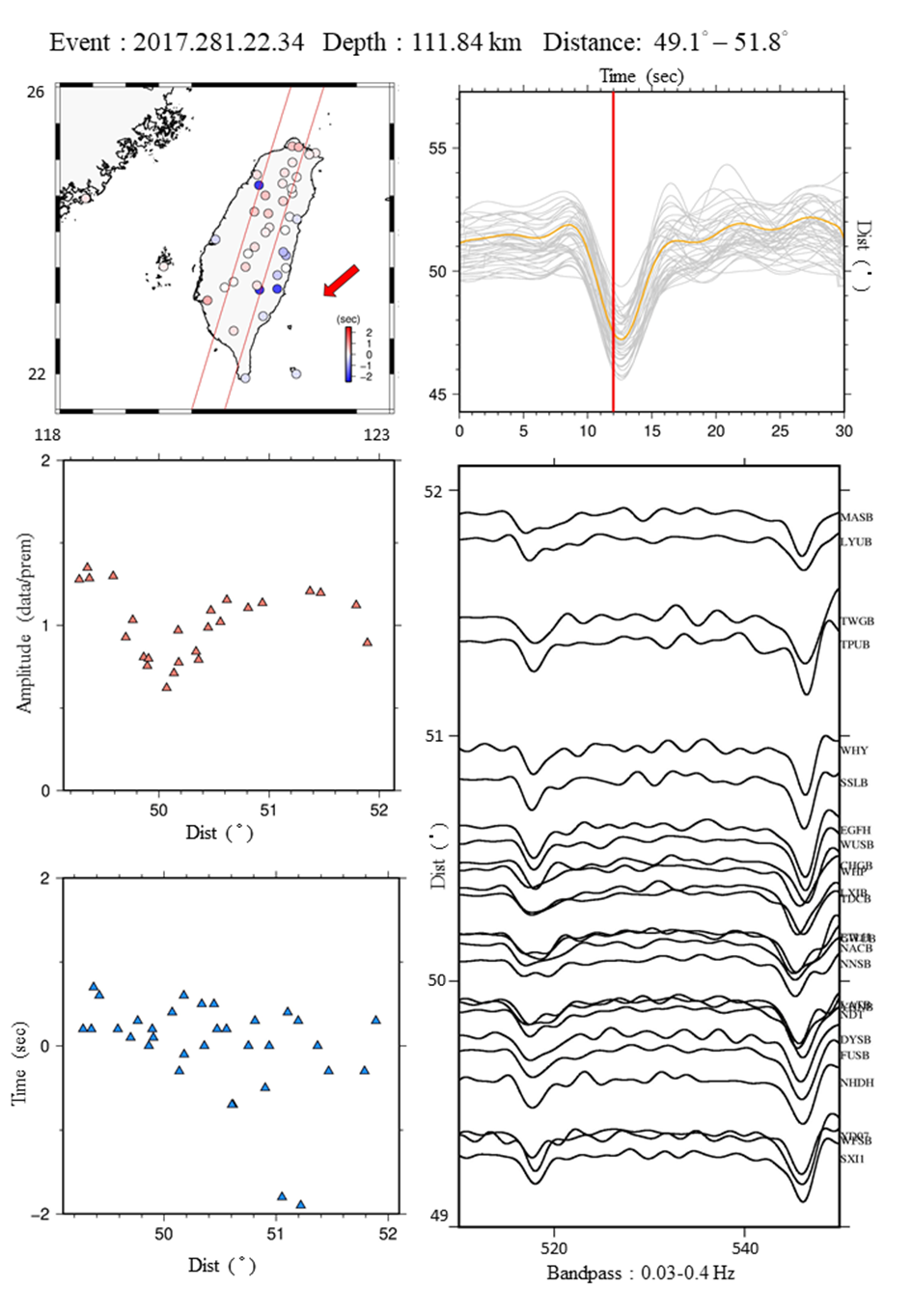

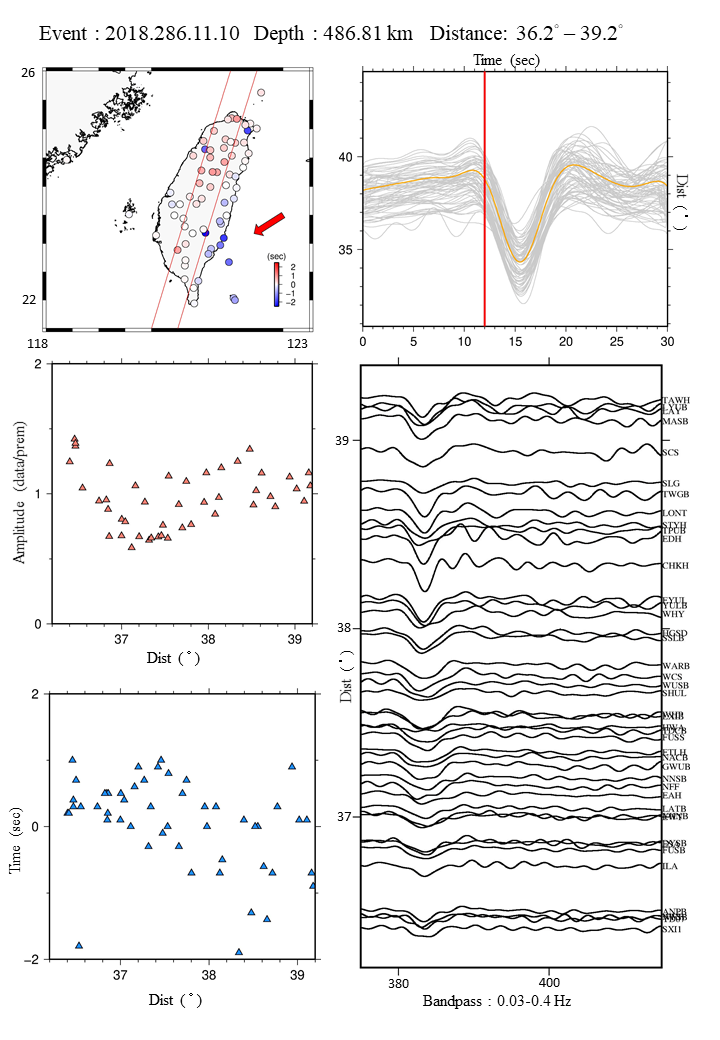


2017/10/08

2018/10/13

2013/10/01

Time (s)

Time (s)

Time (s)

**Extended Data Figure 18 |** Waveform sections for the earthquakes from the north-northeast of Taiwan listed in Table S1. The waveforms were deconvolved to remove the instrument response and subjected to band-pass filtering with cutoff frequencies of 0.03 to 0.4 Hz


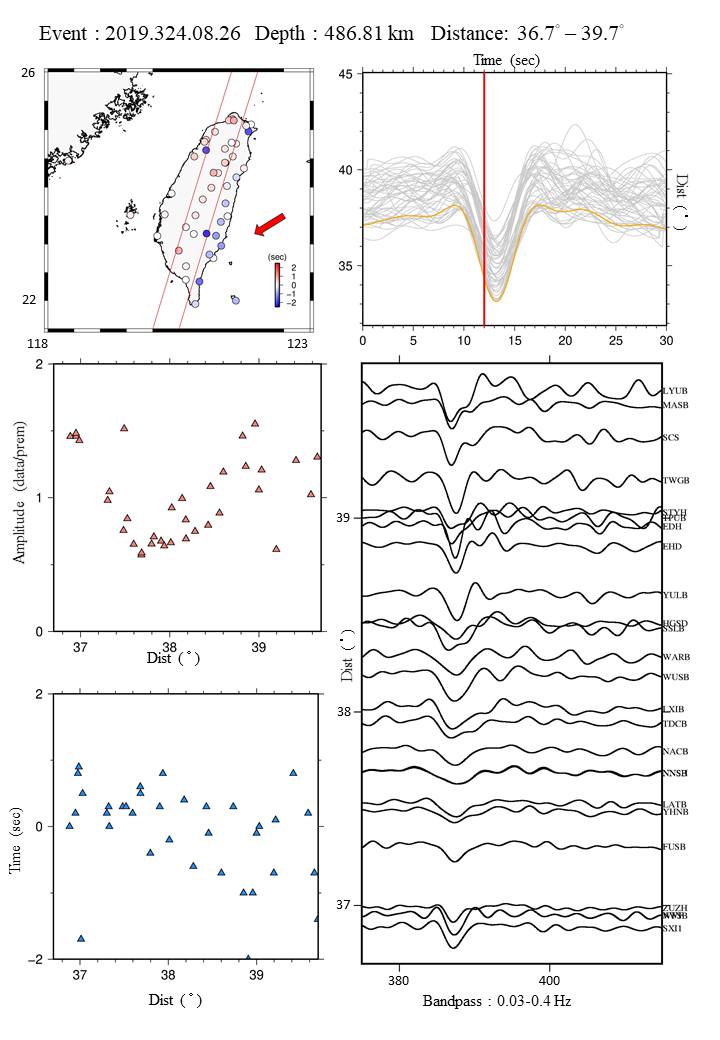

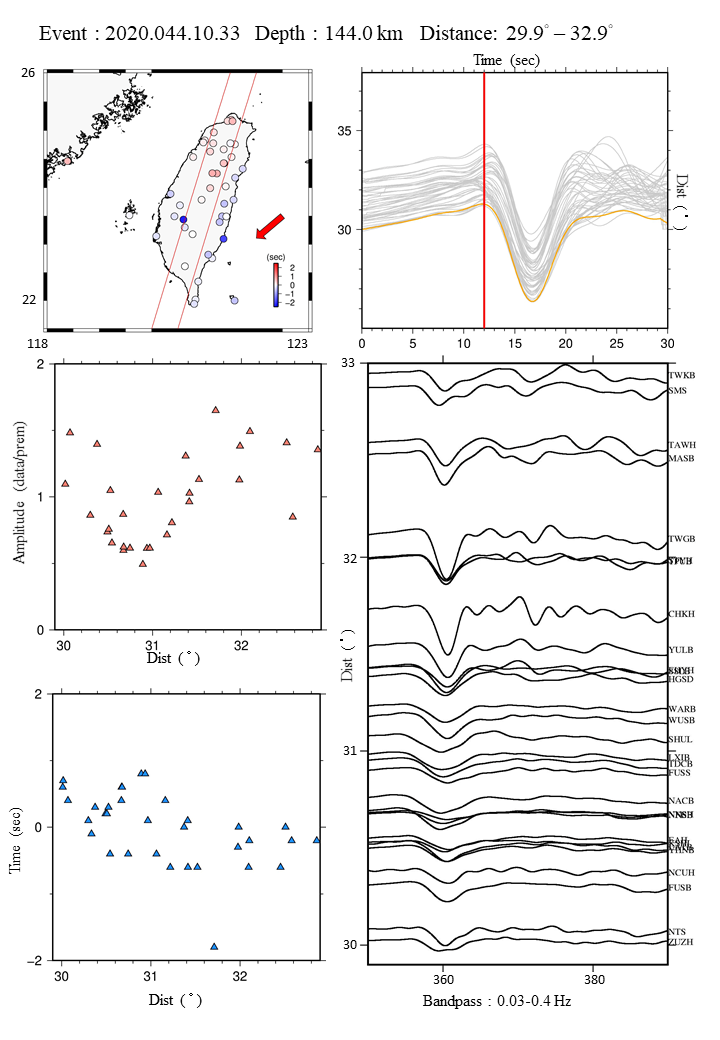

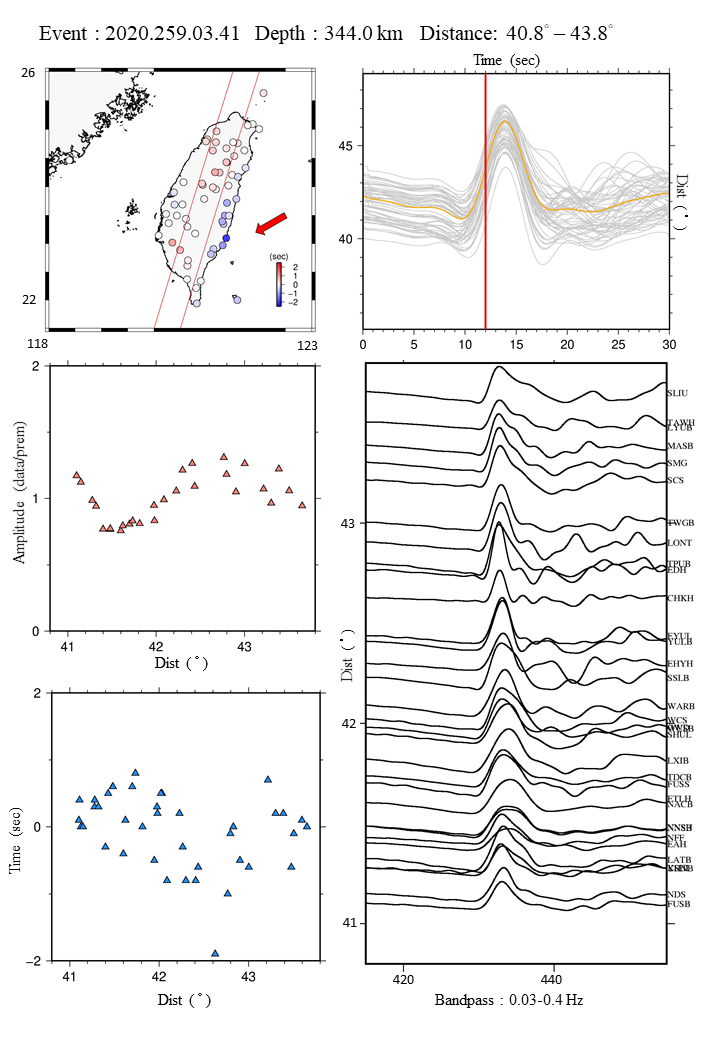


2020/02/13

2020/09/15

2019/11/20

Time (s)

Time (s)

Time (s)

**Extended Data Figure 19 |** Waveform sections for the earthquakes from the north-northeast of Taiwan listed in Table S1. The waveforms were deconvolved to remove the instrument response and subjected to band-pass filtering with cutoff frequencies of 0.03 to 0.4 Hz.


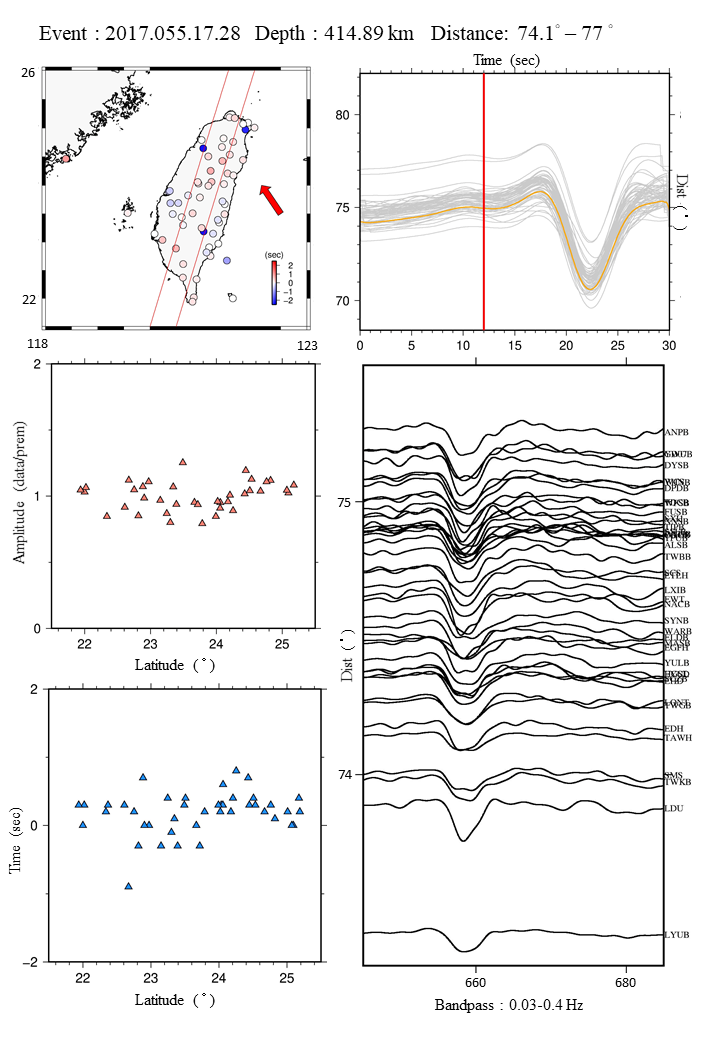

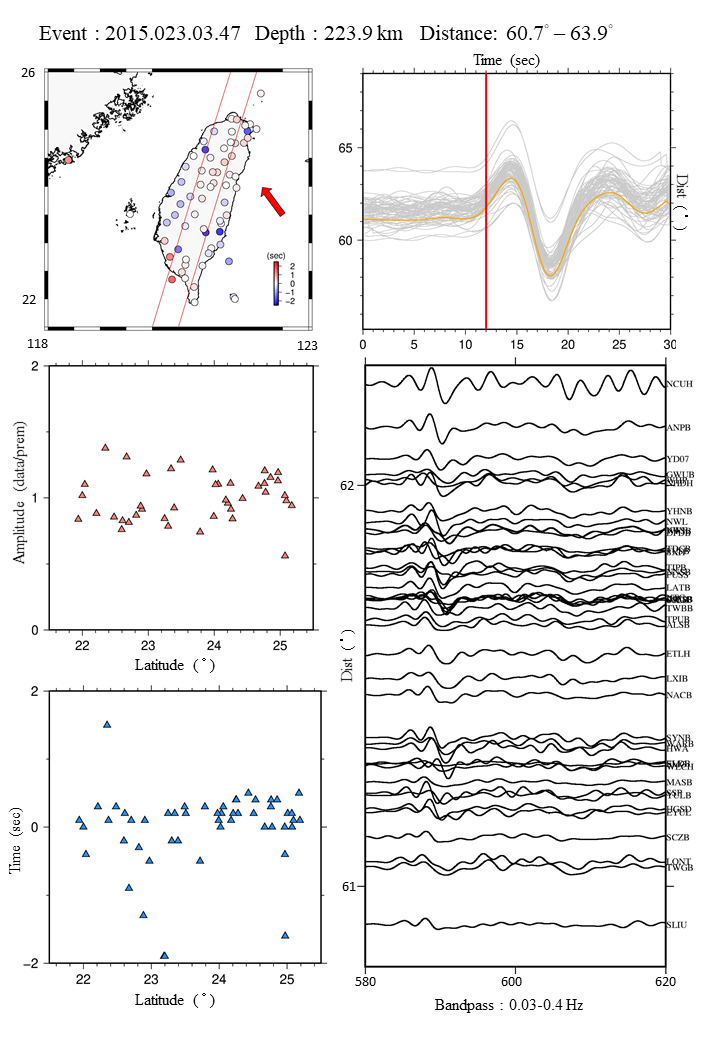

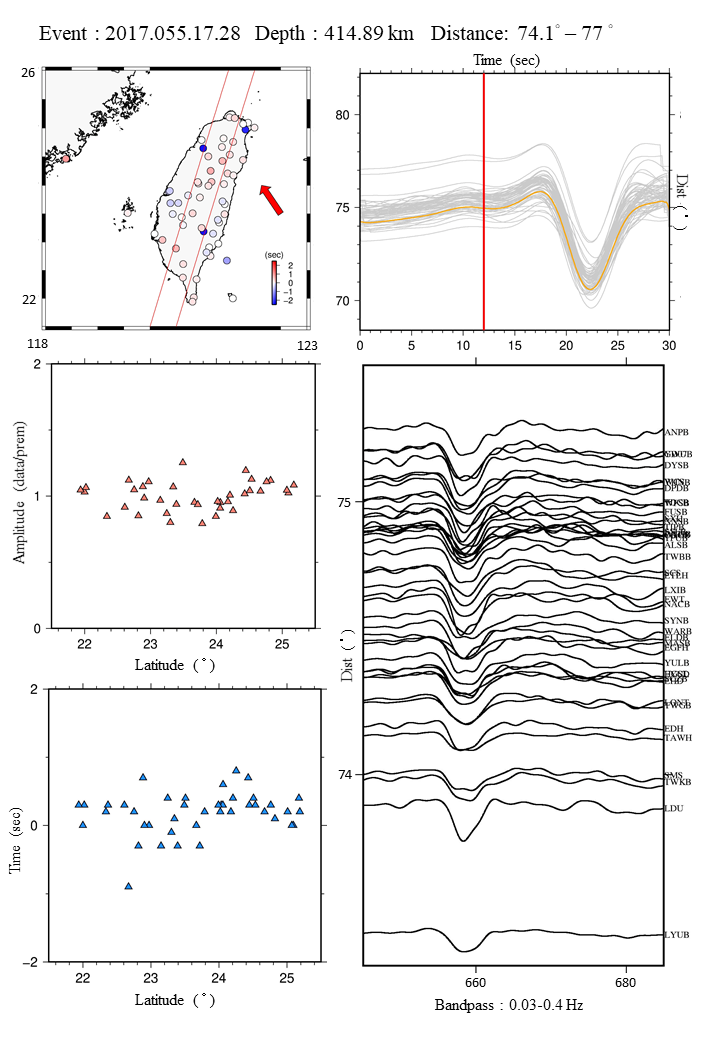

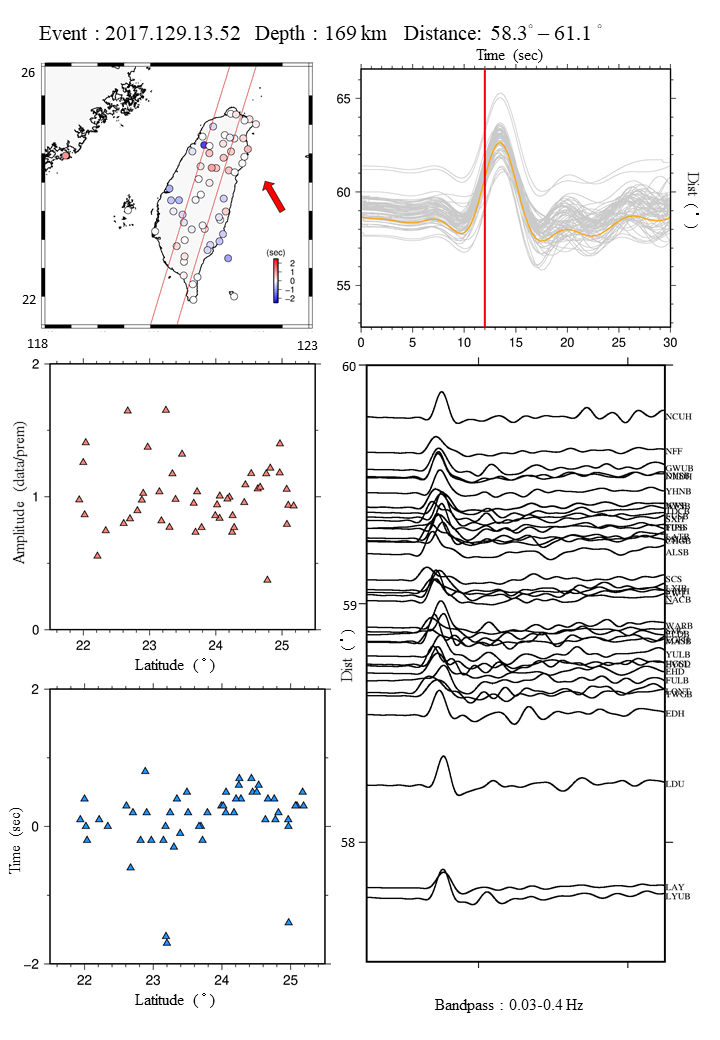


2015/01/23

2017/02/24

2017/05/09


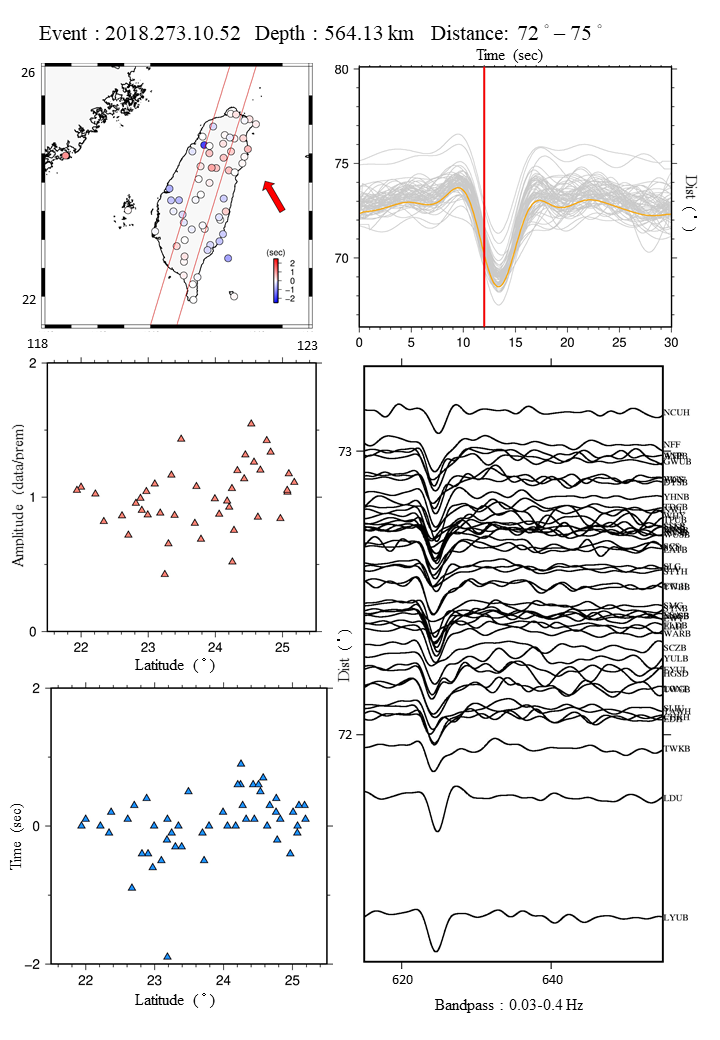

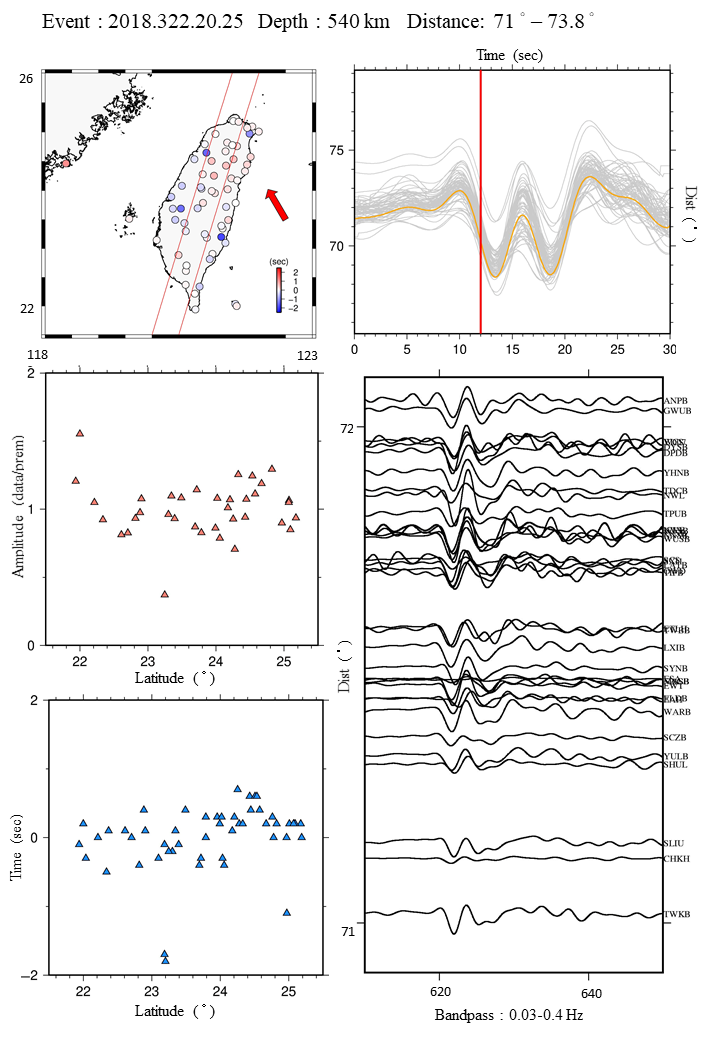

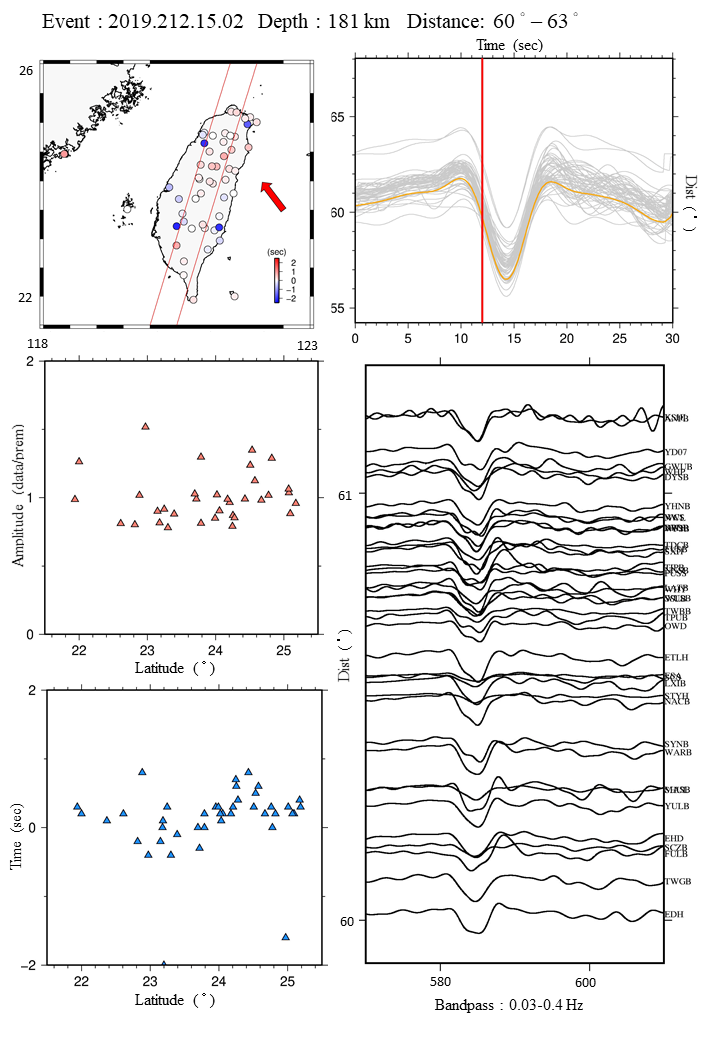


2018/09/30

2018/11/18

2019/07/13

Time (s)

Time (s)

Time (s)

**Extended Data Figure 20 |** Waveform sections for the earthquakes from the southeast of Taiwan listed in Table S1. The waveforms were deconvolved to remove the instrument response and subjected to band-pass filtering with cutoff frequencies of 0.03 to 0.4 Hz.


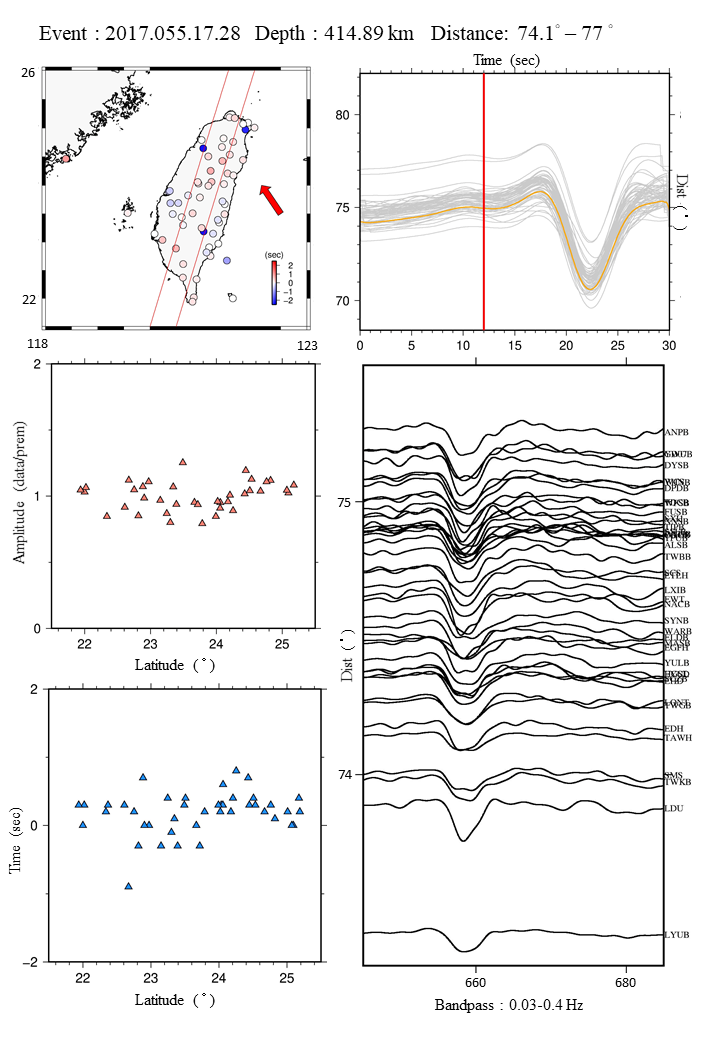

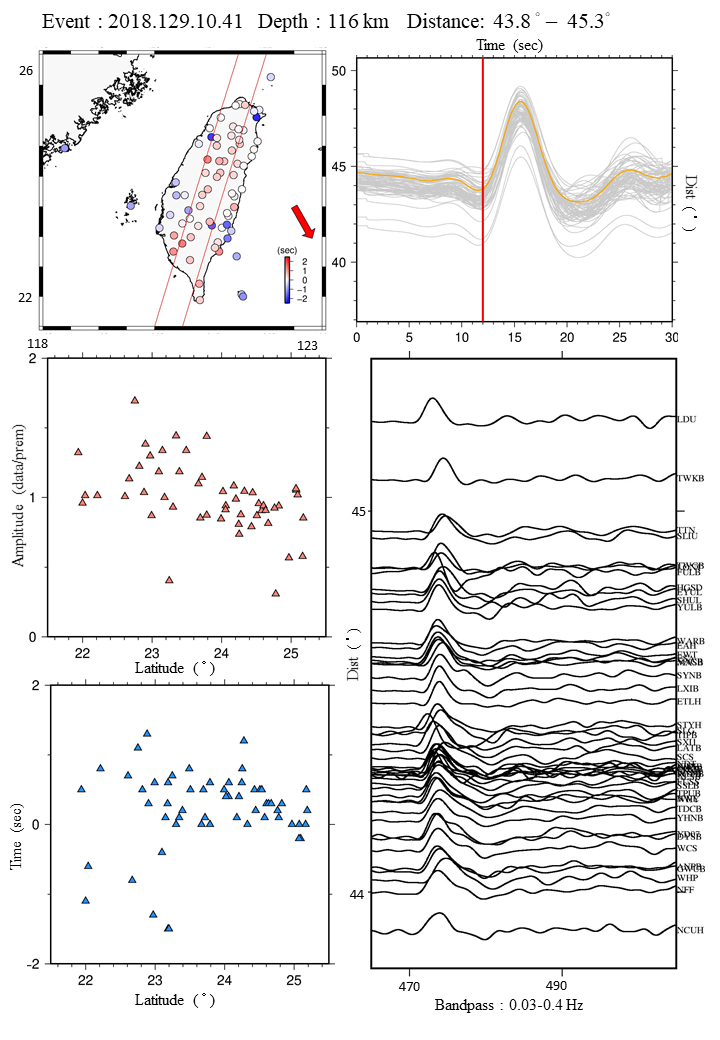


2016/04/10

2018/05/09

Time (s)

Time (s)

**Extended Data Figure 21 |** Waveform sections for the earthquakes from the west of Taiwan listed in Table S1. The waveforms were deconvolved to remove the instrument response and subjected to band-pass filtering with cutoff frequencies of 0.03 to 0.4 Hz.
